# Crystal structures of distinct parallel and antiparallel DNA G-quadruplexes reveal structural polymorphism in *C9orf72* G4C2 repeats

**DOI:** 10.1093/nar/gkaf879

**Published:** 2025-09-10

**Authors:** Yanyan Geng, Changdong Liu, Haitao Miao, Monica Ching Suen, Yuanyuan Xie, Bingchang Zhang, Wanhong Han, Caiming Wu, Haixia Ren, Xueqin Chen, Hwan-Ching Tai, Zhanxiang Wang, Guang Zhu, Qixu Cai

**Affiliations:** Clinical Research Institute of the First Affiliated Hospital of Xiamen University, Fujian Key Laboratory of Brain Tumors Diagnosis and Precision Treatment, Xiamen Key Laboratory of Brain Center, the First Affiliated Hospital of Xiamen University, School of Public Health, School of Medicine, Xiamen University, Xiamen 361003, Fujian, China; Institute for Advanced Study and State Key Laboratory of Molecular Neuroscience, Division of Life Science, The Hong Kong University of Science and Technology, Clear Water Bay, Kowloon, Hong Kong SAR, China; HKUST Shenzhen Research Institute, Hi-Tech Park, Nanshan, Shenzhen 518057, Guangdong, China; Institute for Advanced Study and State Key Laboratory of Molecular Neuroscience, Division of Life Science, The Hong Kong University of Science and Technology, Clear Water Bay, Kowloon, Hong Kong SAR, China; Institute for Advanced Study and State Key Laboratory of Molecular Neuroscience, Division of Life Science, The Hong Kong University of Science and Technology, Clear Water Bay, Kowloon, Hong Kong SAR, China; Department of Neurosurgery and Department of Neuroscience, Fujian Key Laboratory of Brain Tumors Diagnosis and Precision Treatment, Xiamen Key Laboratory of Brain Center, the First Affiliated Hospital of Xiamen University, School of Medicine, Xiamen University, Xiamen 361003, Fujian, China; Department of Neurosurgery and Department of Neuroscience, Fujian Key Laboratory of Brain Tumors Diagnosis and Precision Treatment, Xiamen Key Laboratory of Brain Center, the First Affiliated Hospital of Xiamen University, School of Medicine, Xiamen University, Xiamen 361003, Fujian, China; Department of Neurosurgery and Department of Neuroscience, Fujian Key Laboratory of Brain Tumors Diagnosis and Precision Treatment, Xiamen Key Laboratory of Brain Center, the First Affiliated Hospital of Xiamen University, School of Medicine, Xiamen University, Xiamen 361003, Fujian, China; State Key Laboratory of Cellular Stress Biology, School of Life Sciences, Xiamen University, Xiamen 361102, Fujian, China; State Key Laboratory of Cellular Stress Biology, School of Life Sciences, Xiamen University, Xiamen 361102, Fujian, China; Clinical Research Institute of the First Affiliated Hospital of Xiamen University, Fujian Key Laboratory of Brain Tumors Diagnosis and Precision Treatment, Xiamen Key Laboratory of Brain Center, the First Affiliated Hospital of Xiamen University, School of Public Health, School of Medicine, Xiamen University, Xiamen 361003, Fujian, China; State Key Laboratory of Vaccines for Infectious Diseases, School of Public Health, Xiamen University, Xiamen 361102, Fujian, China; Department of Neurosurgery and Department of Neuroscience, Fujian Key Laboratory of Brain Tumors Diagnosis and Precision Treatment, Xiamen Key Laboratory of Brain Center, the First Affiliated Hospital of Xiamen University, School of Medicine, Xiamen University, Xiamen 361003, Fujian, China; Institute for Advanced Study and State Key Laboratory of Molecular Neuroscience, Division of Life Science, The Hong Kong University of Science and Technology, Clear Water Bay, Kowloon, Hong Kong SAR, China; HKUST Shenzhen Research Institute, Hi-Tech Park, Nanshan, Shenzhen 518057, Guangdong, China; State Key Laboratory of Vaccines for Infectious Diseases, School of Public Health, Xiamen University, Xiamen 361102, Fujian, China

## Abstract

The abnormal expansion of GGGGCC (G4C2) repeats in the noncoding region of the *C9orf72* gene is a major genetic cause of two devastating neurodegenerative disorders, amyotrophic lateral sclerosis (ALS) and frontotemporal dementia (FTD). These G4C2 repeats are known to form G-quadruplex (G4) structures, which are hypothesized to contribute to disease pathogenesis. Here, we demonstrated that four DNA G4C2 repeats can fold into two structurally distinct G4 conformations: a parallel and an antiparallel topology. The high-resolution crystal structure of the parallel G4 reveals an eight-layered dimeric assembly, formed by two identical monomeric units. Each unit contains four stacked G-tetrads connected by three propeller CC loops and is stabilized through 5′-to-5′ π–π interactions and coordination with a central K^+^ ion. Notably, the 3′-ending cytosines form a C·C^+^·C·C^+^ quadruple base pair stacking onto the adjacent G-tetrad layer. In contrast, the antiparallel G4 adopts a four-layered monomeric structure with three edgewise loops, where the C6 and C18 bases engage in stacking interaction with neighboring G-tetrad via a K^+^ ion. These structurally distinct G-quadruplexes provide mechanistic insights into *C9orf72*-associated neurodegeneration and offer potential targets for the development of structure-based therapeutic strategies for ALS and FTD.

## Graphical Abstract

**Graphical Abstract:**
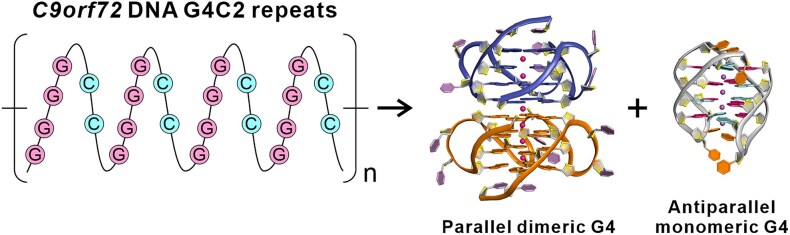

## Introduction

Amyotrophic lateral sclerosis (ALS) and frontotemporal dementia (FTD) are two fatal neurodegenerative disorders that share overlapping pathological and genetic features [1, 2]. ALS is primarily characterized by the degeneration of motor neurons, leading to progressive muscle weakness, atrophy, and eventual death from respiratory failure [3]. In contrast, FTD involves progressive damage to the frontal and temporal lobes of the brain, resulting in significant cognitive and behavioral changes [4, 5]. Currently, ALS and FTD are recognized as part of a broad neurodegenerative continuum that shares common neurodegenerative features [4, 5]. Genetically, familial ALS and FTD are linked by the abnormal expansion of the G4C2 hexanucleotide repeat in the first intron region of the chromosome 9 open reading frame 72 (*C9orf72*) gene [6–8].

Recent progress has focused on understanding the G4C2 repeat expansions in the *C9orf72* gene, particularly their ability to form G-quadruplex (G4) structures—noncanonical four-stranded nucleic acid conformations composed of guanine-rich DNA or RNA sequences [9–11]. The fundamental structural unit of G4s, known as the G-tetrad, is composed of four guanine bases arranged in a square planar structure via cyclic Hoogsteen hydrogen bonding [12]. Two or more G-tetrads can stack on top of one another, stabilized by monovalent cations such as K^+^, to form higher-order G4 assemblies [13]. Notably, G4C2 repeat expansions are highly polymorphic, capable of folding into diverse topologies, depending on the length of the G4C2 repeats and environmental conditions [14–18]. This structural polymorphism of G4s is believed to play a critical role in the pathogenesis of ALS and FTD, by contributing to multiple cellular dysfunctions [11, 19].

Over the past decades, multiple biophysical approaches, including circular dichroism (CD), nuclear magnetic resonance (NMR) spectroscopy, and X-ray crystallography, have been employed to investigate the structural diversity of G4C2 repeat sequences [14, 17, 20–22]. Our earlier work demonstrated that a sequence with four G4C2 repeats, d(G4C2)_4_, the minimal unit capable of forming an intramolecular G4, can fold into a monomeric chair-type antiparallel structure [14]. In the course of the study, other studies revealed that this sequence could adopt alternative antiparallel topologies under different conditions such as pH and cooling rates, further emphasizing the structural polymorphism of these repeats [15, 18]. Additionally, we also previously identified and characterized two novel eight-layer parallel tetrameric G4 structures formed by two G4C2 repeats, suggesting that higher-order G4 assemblies can arise from oligomerization [20, 21]. These findings underscore the intricate structural plasticity of G4C2 repeat sequences and their potential implications in *C9orf72*-associated neurodegenerative diseases [19].

In the present study, we isolated and characterized two distinct G4 topologies formed by four-repeat G4C2 DNA (d(G4C2)_4_): a dimeric parallel form (d(G4C2)_4_-para) and a monomeric antiparallel form (designated d(G4C2)_4_-anti). Using anion exchange chromatography, we successfully separated these conformations and subsequently solved their high-resolution crystal structures. Intriguingly, the crystal structure of d(G4C2)_4_-para reveals a dimeric parallel-stranded G4 comprising eight stacked G-tetrad layers. Each individual d(G4C2)_4_ oligonucleotide folds into a monomeric G4 composed of four G-tetrads, which then dimerize via 5′–5′ π–π stacking interactions, stabilized by a centrally coordinated K^+^ ion. Notably, the 3′-terminal cytosine residues from the two monomers form an intermolecular C·C^+^·C·C^+^ quadruple base pair, which stacks directly onto the adjacent G-tetrad, representing a unique structural feature. In contrast, the d(G4C2)_4_-anti structure adopts a monomeric antiparallel G4 fold composed of four G-tetrads and three edgewise loops. Cytosine residues C6 and C18 align parallel to the neighboring G-tetrads, engaging in π-stacking interactions mediated by an additional K^+^ ion. Together, these findings not only expand the repertoire of *C9orf72* G4C2 G4 folding structures and topologies, but also provide new mechanistic insights into the conformational plasticity of G4C2 repeats and lay a structural foundation for the rational design of therapeutic agents targeting *C9orf72*-linked ALS and FTD.

## Materials and methods

### Sample preparation

Single-stranded DNA oligonucleotides were purchased from Accurate Biotechnology (Hunan, China) and General Biol (Anhui, China). The oligonucleotide was dissolved in a buffer containing 70 mM KCl and 20 mM potassium phosphate (pH 7.0) to a final concentration of 0.1 mM (single strand). The samples were then annealed by heating to 95°C for 20 min followed by slow cooling to room temperature overnight.

For crystallographic studies, DNA samples were further purified using fast protein liquid chromatography (FPLC) using a Mono Q column (Cytiva). The binding buffer consisted of 70 mM KCl and 20 mM potassium phosphate (pH 7.0), while the elution buffer contained 1 M KCl and 20 mM potassium phosphate (pH 7.0). Gradual elution with increasing KCl concentration separated different G4 conformers and non-specific fractions. Desired fractions were collected, concentrated, and buffer-exchanged into 20 mM Tris–HCl, 100 mM KCl (pH 7.0) for crystallization.

### NMR spectroscopy

NMR experiments were performed at 25°C on 850 MHz Bruker spectrometers. DNA samples were prepared at a concentration of ∼0.1 mM (single strand) in 20 mM potassium phosphate buffer with 70 mM KCl (pH 7.0).

### CD spectroscopy

CD spectra were recorded using a JASCO J-1700 spectropolarimeter at 25°C. Measurements were conducted in a 1 mm path-length quartz cuvette with a sample volume of 400 μl. DNA oligonucleotides were prepared at a concentration of 15 μM (single strand) in 20 mM potassium phosphate buffer containing 70 mM KCl (pH 7.0).

### CD melting

The CD melting experiments were performed with a temperature range from 25°C to 95°C at 1°C/min. The concentration of oligonucleotides was at 20 μM for the single strand. The CD signal was measured at a single wavelength and then normalized by using the equation (CD signal − min)/(max − min), in which CD signal is the absorbance at a given temperature. The max is the maximum absorbance at 260 nm (for parallel G4s) or 290 nm (for antiparallel/hybrid G4s), and min is the minimum value. Data were fitted by the Boltzmann sigmoid equation or double sigmoidal equation (GraphPad Prism).

### Polyacrylamide gel electrophoresis

Non-denaturing polyacrylamide gel electrophoresis (PAGE) was carried out in 20% polyacrylamide gels (acrylamide:bis-acrylamide ratio 29:1), supplemented with 20 mM KCl in both the gel and 0.5 × TBE running buffer. Samples were prepared at a concentration of 100 μM (single strand). Gels were stained using GelRed dye (Biotium).

### Size exclusion chromatography coupled with multi-angle light scattering

The size exclusion chromatography coupled with multi-angle light scattering (SEC–MALS) system consists of an HPLC system (Agilent), a static light scattering detector (Wyatt), and a differential refractive index detector (Agilent). A 100 μl sample of d(G4C2)_4_-para or d(G4C2)_4_-anti with the concentration of 200 μM was loaded by autosampler (Agilent) into a Superose 12 10/300 column pre-equilibrated with the buffer containing 70 mM KCl and 20 mM potassium phosphate (pH 7.0). Data were analyzed by ASTRA7 (Wyatt).

### Crystallization

The d(G4C2)_4_-para sample was concentrated to 1.8 mM and initially screened for crystallization using the Natrix HT kit (Hampton Research) with the sitting-drop vapor-diffusion method at 16°C. After 2 months, football-shaped crystals were obtained from a condition containing 0.1 M KCl, 0.05 M sodium cacodylate trihydrate (pH 6.0), 16% (w/v) polyethylene glycol (PEG)-1000, and 0.0005 M spermine.

The d(G4C2)_4_-anti sample was concentrated to 2.5 mM and screened using the Helix Screen (Molecular Dimensions) under similar sitting-drop vapor-diffusion conditions at 16°C. After several weeks, thin slice-shaped crystals were obtained from a well containing 0.05 M KCl, 0.05 M BIS-TRIS (pH 7.0), 33% (w/v) PEG-3350, and 0.005 M spermine.

### Data collection and structure determination

For data collection, the crystals of d(G4C2)_4_-para and d(G4C2)_4_-anti were cryoprotected by 25% PEG400 and flash-cooled in liquid nitrogen. The diffraction data sets were collected on beamlines BL19U1 and BL02U1 at Shanghai Synchrotron Radiation Facility (SSRF) at the wavelengths as indicated in Table 1. Intensity data were integrated and scaled by XDS [23]. The structures of d(G4C2)_4_-para and d(G4C2)_4_-anti were solved by molecular replacement method by Phaser [24] using d(G4C2)_2_ (PDB: 7ECG) and d[(G4C2)_3_G4]-Br-G21 (PDB: 2N2D) as the searching models, respectively. Manual model building and refinement were performed iteratively with COOT [25], Refmac5 [26], and Phenix.refine [27]. The parameters of refinement were optimized by PDB-REDO [28]. The final refinement statistics were summarized in Table 1. All figures of G4 structures were prepared using PyMOL (http://www.pymol.org).

**Table 1.** Crystallographic data collection and refinement statistics

Data collection	d(G4C2)_4_-para	d(G4C2)_4_-anti
Space group	I4_1_	P2_1_2_1_2_1_
Wavelength (Å)	0.91 776	0.919 764
Unit cell parameters	a = b = 64.036 Å c = 33.293 Å α=β=γ=90°	a = 31.288 Å b = 87.147 Å c = 98.302 Å α=β=γ=90°
Resolution range (Å)	50–1.94 (2.06–1.94)	50–2.70 (2.86–2.70)
No. of unique reflections	5104 (809)	7881 (1240)
Redundancy	12.8 (13.5)	9.8 (8.9)
I/σ	12.16 (1.14)	12.12 (1.74)
Completeness (%)	99.8 (99.4)	99.8 (99.2)
R_merge_^a^ (%)	15.0 (254.1)	16.0 (157.7)
CC_1/2_	0.999 (0.507)	1.000 (0.335)
Structure refinement		
Resolution (Å)	1.94	2.70
R_work_^b^ (%)	24.22	23.34
R_free_^c^ (%)	28.38	25.16
RMSD bonds (Å)	0.003	0.005
RMSD angles (°)	1.394	1.894
Average B factor (Å^2^)	47.4	66.5
No. of atoms		
DNA	501	1894
Water	23	6
Ion	4	16
B factor (Å^2^)		
DNA	47.8	66.7
Water	42.2	36.8
Ion	23.2	50.1

Numbers in parentheses represent the values for the highest-resolution shell.

^a^R_merge_ = Σ|*I*_i_ − |/Σ*I*_i_, where *I*_i_ is the intensity of measured reflection and is the mean intensity of all symmetry-related reflections.

^b^R_work_ = Σ_W_||*F*_calc_| − |*F*_obs_||/Σ|*F*_obs_|, where *F*_obs_ and *F*_calc_ are observed and calculated structure factors. W is working dataset of ∼95% of the total unique reflections randomly chosen and used for refinement.

^c^R_free_ = Σ_T_||*F*_calc_| − |*F*_obs_||/Σ|*F*_obs_|, where T is a test dataset of ∼5% of the total unique reflections randomly chosen and set aside prior to refinement.

## Results

### d(G4C2)_4_ forms parallel and antiparallel G4s in K^+^ solution

**Figure 1.**
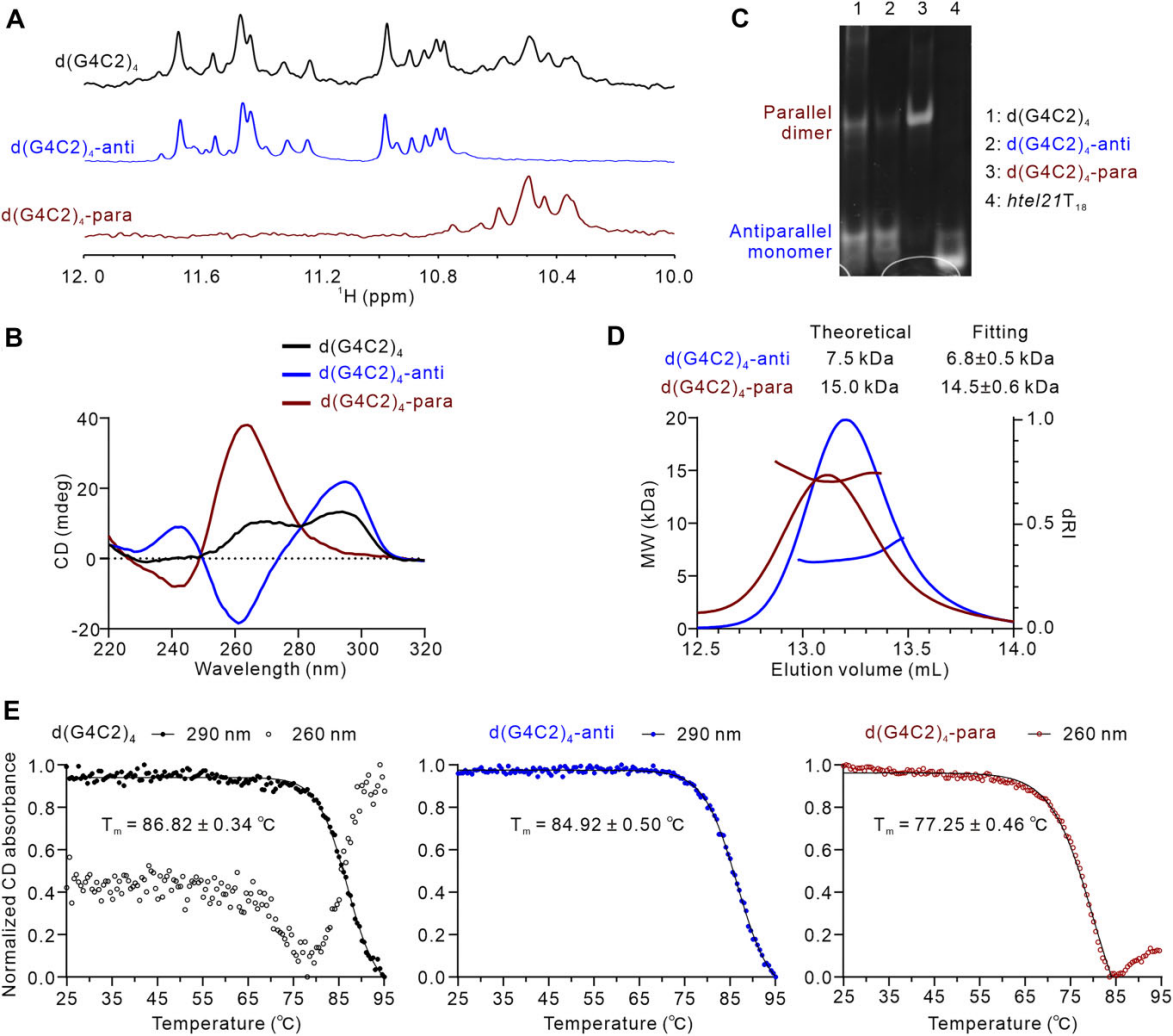
Characterization of d(G4C2)_4_, d(G4C2)_4_-para, and d(G4C2)_4_-anti separated by anion exchange chromatography in K^+^ solutions. (**A**) The imino region of 1D ^1^H-NMR spectra in 20 mM potassium phosphate solution containing 70 mM KCl with pH 7.0 recorded at 25°C on 850 MHz. (**B**) CD spectra in 20 mM potassium phosphate solution containing 70 mM KCl with pH 7.0 recorded at 25°C. (**C**) Electrophoretic mobility in 20% native PAGE at 100 μM concentration with a reference of d *htel21*T_18_ (d[(GGGTTA)_2_GGGTTTGGG]), which is a monomeric three-layer antiparallel G4 [30]. (**D**) The SEC–MALS assay of d(G4C2)_4_-para and d(G4C2)_4_-anti in the buffer containing 70 mM KCl, 20 mM potassium phosphate (pH 7.0). The fitted molecular weights are expressed as the best fitted values ± SE. (**E**) CD melting curves in 20 mM potassium phosphate solution containing 70 mM KCl with pH 7.0. Data were fitted by the Boltzmann sigmoid equation (GraphPad Prism). The *T*_m_ value reported for para-d(G4C2)_4_ was determined from the data within the 25°C to 85°C range.

Previous studies have shown that d(G4C2)_4_ can fold into an antiparallel G4 in K^+^ solution, as revealed by NMR and spectroscopic analyses [14, 15, 18]. This structure corresponds to the peaks with strong signals located between 11.0 and 12.0 ppm in the 1D ^1^H NMR spectrum of d(G4C2)_4_ (Fig. 1A). Interestingly, several broad peaks with lower intensity are also observed in the region of 10.5–11.0 ppm. Notably, the CD spectrum of d(G4C2)_4_ exhibits features of a typical hybrid G4 conformation, characterized by two dominant positive peaks at ∼264 and ∼295 nm along with a trough around 240 nm (Fig. 1B) [29]. Considering the structural polymorphism of G4s and to investigate whether the minor peaks represent an unveiled G4 structure formed by d(G4C2)_4_, we performed further purification using Mono Q anion exchange chromatography. This resulted in the separation of two distinct fractions (Supplementary Fig. S1). Remarkably, CD spectra revealed that one fraction exhibits a typical parallel G4 signature, with a dominant positive peak at ∼260 nm and a negative peak at ∼240 nm, referred to as d(G4C2)_4_-para (Fig. 1B). As expected, the other fraction shows a positive peak at ∼295 nm and a negative peak at ∼260 nm, consistent with previously reported antiparallel G4 form, named d(G4C2)_4_-anti (Fig. 1B). Both d(G4C2)_4_-para and d(G4C2)_4_-anti yielded well-resolved 1D ^1^H NMR spectra, indicating the formation of distinct well-folded G4 structures (Fig. 1A). Intriguingly, the parallel G4s formed by both d(G4C2)_2_ and d(G4C2)_4_ exhibited a similar imino proton chemical shift region, ranging from ∼10.6 to ∼11.2 ppm (Supplementary Fig. S2A). Additionally, the NMR spectrum of NAN, d[(G4C2)_3_GGBrGG] [15], displayed the imino proton resonances within ∼11.0–12.0 ppm, consistent with the d(G4C2)_4_-anti characterized here.

Subsequently, we performed native PAGE to assess the molecular sizes of d(G4C2)_4_-para and d(G4C2)_4_-anti in K^+^ solution, using *htel21*T_18_ (a 21-nt monomeric antiparallel G4 with three tetrads) as a reference [30]. As shown in Fig. 1C, d(G4C2)_4_-anti migrated similarly to *htel21*T_18_, indicating a monomeric structure. In contrast, the migration of d(G4C2)_4_-para is slower than d(G4C2)_4_-anti, suggesting the formation of a multimeric assembly. Notably, native PAGE of d(G4C2)_4_ prior to anion exchange chromatography revealed two bands, corresponding to d(G4C2)_4_-para and d(G4C2)_4_-anti, respectively. To further validate their assembly, we performed SEC–MALS. The measured molecular weight of d(G4C2)_4_-para was 14.5 ± 0.6 kDa, closely matching the theoretical molecular weight of a dimer (15.0 kDa), indicating the formation of a dimeric G4 in solution (Fig. 1D). In contrast, d(G4C2)_4_-anti had a molecular weight of 6.8 ± 0.5 kDa, which corresponds well with the theoretical monomeric molecular weight (7.5 kDa) (Fig. 1D). These findings are consistent with previous structural studies [15, 18] and support the formation of both parallel dimeric and antiparallel monomeric G4 structures by d(G4C2)_4_ under physiological K^+^ conditions.

### Stability of G4s formed by d(G4C2)_n_ investigated by CD melting experiments

Both d(G4C2)_4_-anti and d(G4C2)_4_-para were stable during purification by anion exchange chromatography (Supplementary Fig. S1) and exhibited monodisperse peaks during SEC–MALS analysis (Fig. 1D), confirming their structural stability. We further assessed the thermal stability of G4s formed by d(G4C2)_4_ by CD melting experiments. Unpurified d(G4C2)_4_ displayed a clear transition at 290 nm with the fitted *T*_*m*_ value of 86.82°C, but the biphasic melting at 260 nm indicated two components in the unpurified d(G4C2)_4_ (Fig. 1E). Purified d(G4C2)_4_-anti and d(G4C2)_4_-para showed distinct stabilities. d(G4C2)_4_-anti (*T*_*m*_ = 84.92°C) exhibited significantly higher thermal stability than d(G4C2)_4_-para (*T*_*m*_ = 77.25°C) (Fig. 1E). The CD melting assays were also performed to evaluate the thermal stability of G4s formed by d(G4C2)_2_, which can fold into a hybrid form (d(G4C2)_2_-hybrid) and a tetrameric parallel form (d(G4C2)_2_-para) [17, 20]. The *T*_*m*_ values were 79.57°C for d(G4C2)_2_-hybrid and 46.38°C/89.07°C for d(G4C2)_2_-para (Supplementary Fig. S2B). Under the current experimental conditions (the purified samples in K^+^ buffer), the monomeric d(G4C2)_4_-anti exhibits higher thermal stability than other known conformations. However, the folding behavior of long G4C2 repeat sequences under native physiological conditions remains to be elucidated, and their topological preferences may differ from the shorter sequences studied here.

### Crystal structure of d(G4C2)_4_-para reveals a dimeric parallel G4

**Figure 2.**
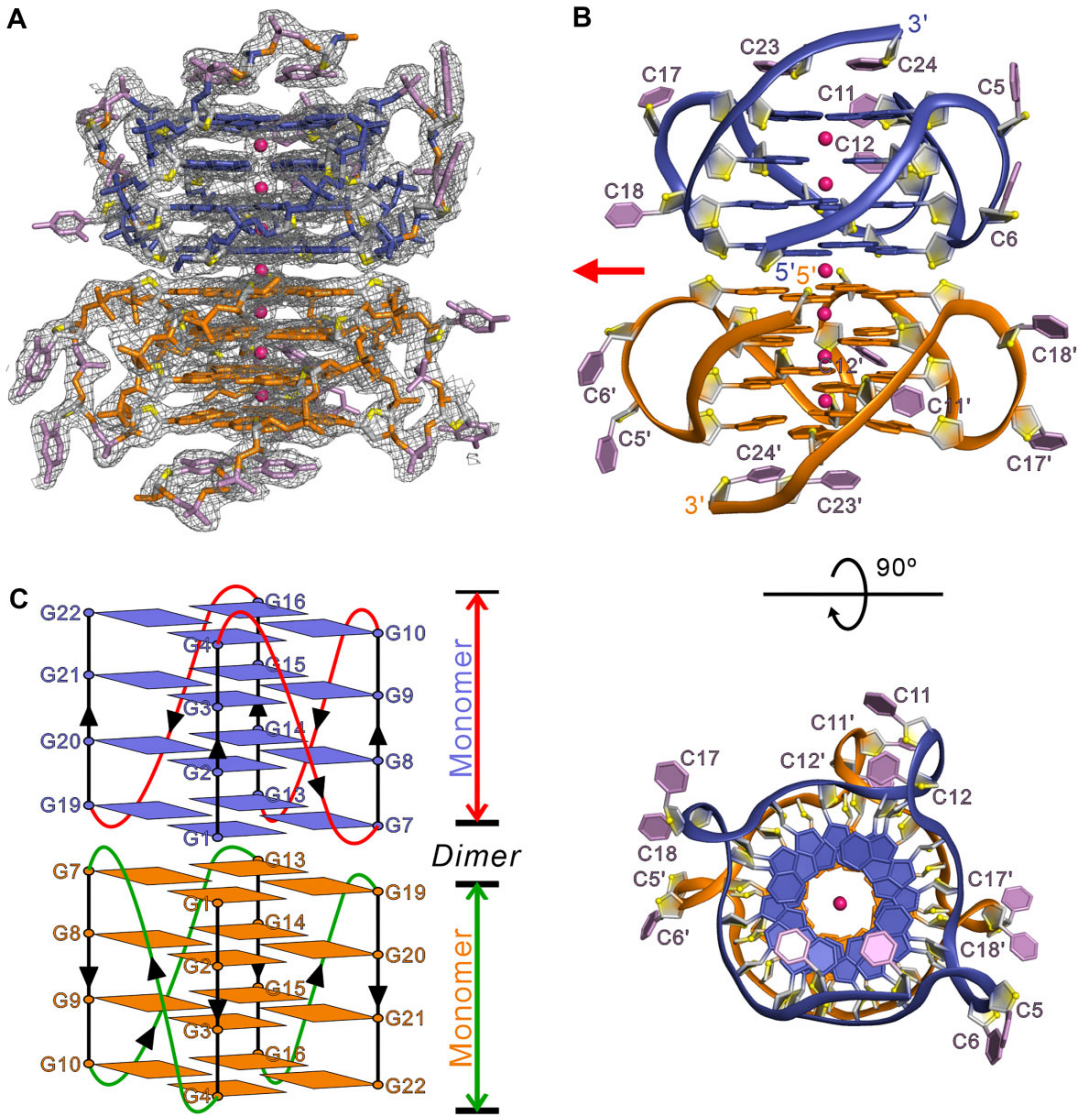
Overall structure of d(G4C2)_4_-para in K^+^ solution. (**A**) The electron density map of the final *2mFo −DFc* map contoured at 1.0 σ and stick representation for d(G4C2)_4_-para in dimeric form. (**B**) Cartoon representation of dimeric parallel G4 formed by d(G4C2)_4_. Each monomeric block is stacked to form a dimeric G4 via a 5′-to-5′ mode, G1-to-G1, stabilized by K^+^. Each molecule, d(G4C2)_4_, is shown as orange and blue in the dimeric G4. O4′ oxygens are in yellow, the cytosine bases are in pink, and potassium ions are in hot pink. The prime (′) notation in the figure signifies the crystallographically symmetric oligonucleotide strand in the crystal. The red arrow indicates the crystallographic two-fold axis of I4_1_ space group. (**C**) Schematic representation of topology adopted by d(G4C2)_4_-para in dimeric form.

**Figure 3.**
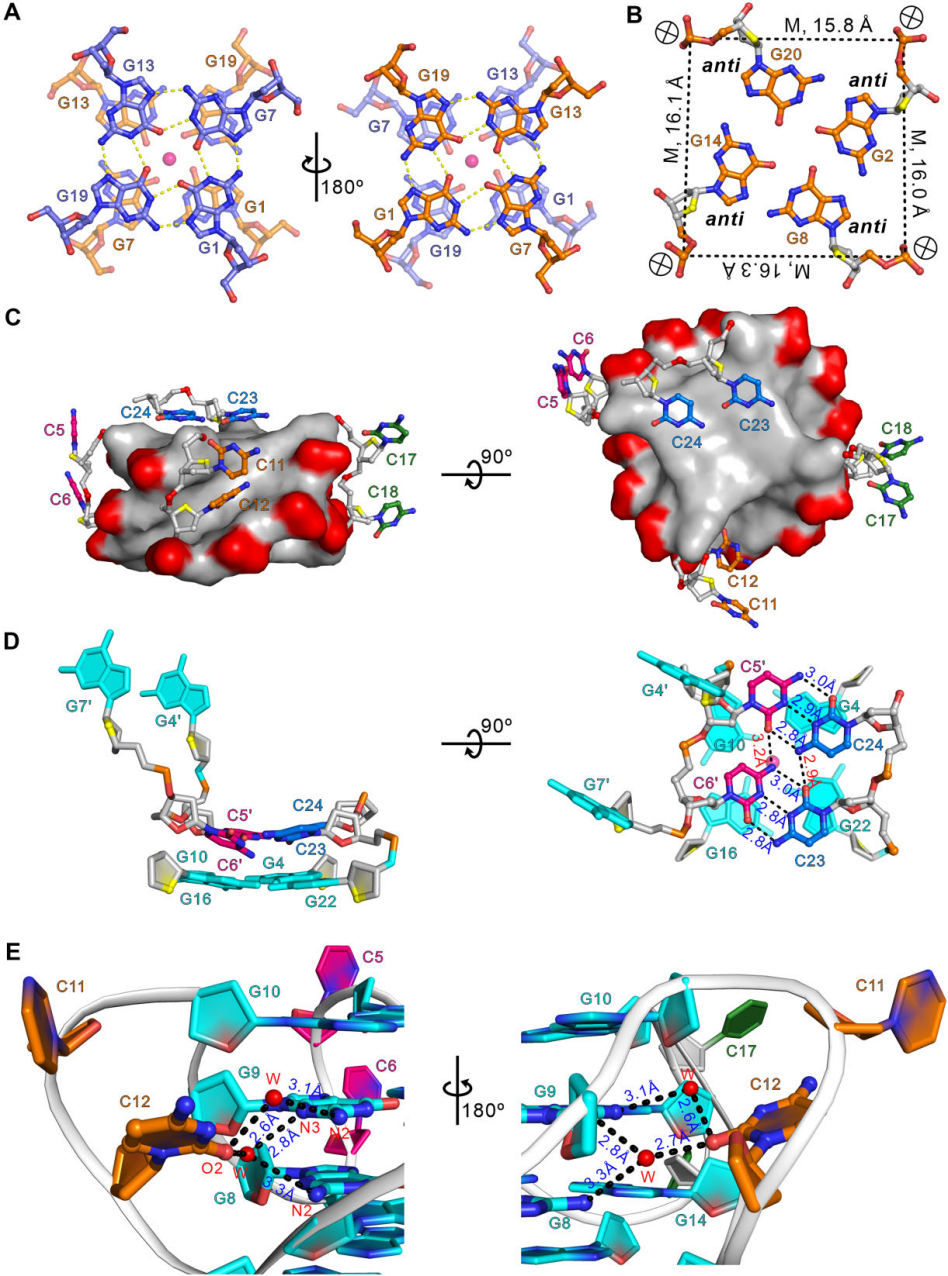
Structural details of d(G4C2)_4_-para. (**A**) G-tetrad stacking mode at the interface of two monomeric G4 blocks in the dimeric crystal structure of d(G4C2)_4_. Hydrogen bonds are depicted as dashed yellow lines. (**B**) Representative view of the medium groove formed by the core G-tetrad G2·G8·G14·G20. Average groove width values are indicated by phosphate–phosphate distances shown as dashed lines. ⊗: 5′→3′ strand direction pointing into the paper. (**C**) Structural arrangement of the three propeller loops comprising C5, C6, C11, C12, C17, and C18, along with the 3′-terminal bases C23 and C24. The G4 core is shown in surface representation. (**D**) Intermolecular and intramolecular π–π stacking interactions involving C5-C6 and C23-C24 bases with the G-tetrad. A unique intermolecular C·C^+^·C·C^+^ quadruple base pair is formed between the C5-C6 and C23-C24 bases. The prime (′) notation in the figure indicates that the bases originate from distinct oligonucleotide strands. (**E**) Conformations of C11 and C12 bases and their interactions with G-tetrad bases, mediated by water molecules. All the hydrogen bonds are depicted as dashed lines. The water molecules are shown as red spheres and K^+^ ions are represented in magenta.

To elucidate the molecular basis underlying the formation of the parallel G4 by d(G4C2)_4_-para, we successfully crystallized this oligonucleotide in the space group of I4_1_ and solved the structure using molecular replacement method at a resolution of 1.94 Å (Table 1 and Fig. 2A). The asymmetric unit contains a single d(G4C2)_4_ oligonucleotide strand. Each d(G4C2)_4_ molecule adopts a parallel-stranded G4 configuration, consisting of four G-tetrads stabilized by three evenly spaced K^+^ ions positioned along the central axis. These tetrads are connected by two CC double-chain-reversal loops (Supplementary Fig. S3A). Intriguingly, two crystallographically symmetric monomeric G4 units stack co-axially via π–π interactions in a 5′-to-5′ orientation (Supplementary Fig. S3B). Specifically, the G1 base from one unit stacks directly onto the G1 base of the symmetry-related monomer across a crystallographic two-fold axis, forming a dimeric G4 structure (Figs 2B and 2C). This dimeric arrangement is consistent with the results of native PAGE (Fig. 1C) and SEC–MALS (Fig. 1D). Notably, the two monomeric units are further stabilized by a well-defined central-channel K^+^ ion located at the 5′-to-5′ stacking interface, which aligns along the same axis as the other six K^+^ ions (Figs 2B and 3A).

In each monomeric unit of d(G4C2)_4_, the hydrogen-bond directionalities of the four G-tetrads follow a clockwise orientation (G1→G7→G14→G9, G2→G8→G14→G20, G3→G9→G15→G21, and G4→G10→16→G22). The Hoogsteen hydrogen bonds, especially between N1-O6 and N2-N7, are oriented from the electron pair acceptor to the electron pair donor. Notably, all guanines based in each G4 adopt the *anti*-glycosidic conformations, resulting in four medium grooves with widths of 15.8/16.0/16.1/16.4 Å, respectively (Fig. 3B). Interestingly, four water clusters were observed within the medium grooves. These water molecules form hydrogen bonds with the N2 and N3 atoms of guanines, the O4′ oxygen of deoxyribose, and the phosphate groups, thereby creating water networks closely associated with the loop regions and contributing to the stabilization of loop conformations (Supplementary Fig. S4).

The oligonucleotide, d(G4C2)_4_-para, contains eight cytosine bases. Among them, C5, C6, C11, C12, C17, and C18 form three propeller loops that interconnect the four G-tetrads, while the two 3′-ending cytosine bases (C23, C24) twist back to stack on the outermost G-tetrad (Figs 2 and 3C). The electron densities are well-defined for most of the cytosine bases, except C11, C17, and C18. Specifically, C5 and C6 bases from a neighboring strand interact with C23 and C24 to form a C·C^+^·C·C^+^ quadruple base pair, which stacks above the outer G-tetrad layer, G4·G10·G16·G22 (Fig. 3D). Two C·C^+^ base pairs are formed by C6′·C23 and C5′·C24, where the cytosine bases are connected via hydrogen bonds: C6′N4-C23O2, C6′N3-C23N3, C6′O2-C23N4, C5′N4-C24O2, C5′N3-C24N3, and C5′O2-C24N4. In addition, the two C·C^+^ base pairs are connected through hydrogen bonds between C6′N4-C5′O2 and C23O2-C24N4 (Fig. 3D) [Here, the prime (′) notation indicates that the bases belong to separate oligonucleotide strands]. Additionally, the O2 atom of C12 is bridged to the N2 atom of G8 and the N2 and N3 atoms of G9 through two structured water molecules (Fig. 3E), effectively inserting C12 into the medium groove (Fig. 3C). The intermolecular interactions of cytosine bases observed in d(G4C2)_4_-para are potentially caused by crystal packing (Fig. 3C–E). However, similar C·C^+^·C·C^+^ quadruple base pairs are also observed in the structure of d(G4C2)_2_ [20], demonstrating these intermolecular interactions may be formed by these sequences in solution.

### Comparison of d(G4C2)_4_-para with other G4s formed by G4C2 repeats

Recently, we have determined the crystal structures of G4s formed by two repeats of DNA and RNA G4C2 sequences, d(G4C2)_2_ and r(G4C2)_2_, respectively [20, 21]. Both structures adopt parallel, propeller-type conformations, assembling into tetrameric G4s composed of two dimeric, four-layered G4 subunits stacked in a 5′-to-5′ orientation, with a coordinated central K^+^ ion. Similarly, d(G4C2)_4_-para folds into an eight-layer parallel G4 architecture consisting of two monomeric G4 units, each comprising four G4C2 repeats (Fig. 2). Intriguingly, the stacking mode in d(G4C2)_4_-para is exclusively of the Form-1/1 type, where the G1 base of one G4 unit stacks directly with the G1 base of its symmetry-related partner. In contrast, d(G4C2)_2_ exhibits both Form-1/1 and Form-1/7 stacking (G1 stacking with G7) [20], whereas r(G4C2)_2_ adopts only the Form-1/7 mode [21]. This highlights the unique stacking arrangement of d(G4C2)_4_-para and underscores the structural polymorphism of G4 assemblies.

Superposition analysis further reveals that d(G4C2)_4_-para is similar to d(G4C2)_2_ and r(G4C2)_2_, with all-heavy-atom root-mean-square deviations (RMSD) of ∼0.430 and ∼0.722 Å over their four-layer G-core, respectively (Supplementary Fig. S5). The propeller loops—C5–C6, C11–C12, and C17–C18—in d(G4C2)_4_-para adopt conformations consistent with those observed in the shorter constructs, either extending outward from the G-core or inserting into the medium groove (Supplementary Fig. S5). The 3′-overhangs (C23 and C24 in d(G4C2)_4_-para, corresponding to C11 and C12 in d(G4C2)_2_ and r(G4C2)_2_) form a C·C^+^·C·C^+^ quadruple base pair, a feature also observed in d(G4C2)_2_. Notably, in r(G4C2)_2_, the two cytosine bases of 3′-overhangs stack parallel to adjacent G-tetrad layers with K^+^ coordination, yet without forming C·C base pairs. Collectively, these comparisons underscore the critical role of loop composition and conformation in modulating G4 stability and diversity.

### A monomeric antiparallel G4 structure formed by d(G4C2)_4_-anti in K^+^ solution

**Figure 4.**
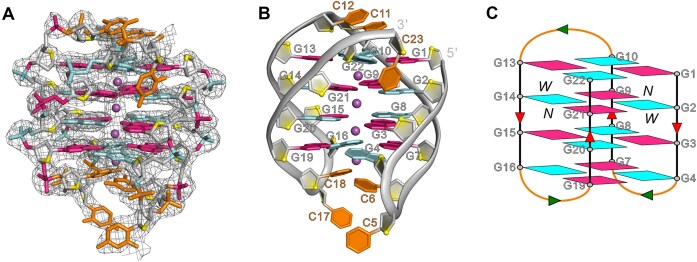
Overall structure of d(G4C2)_4_-anti in K^+^ solution. (**A**) The electron density map of the final *2mFo*−*DFc* map contoured at 1.0 σ for d(G4C2)_4_-anti. (**B**) Cartoon representation of monomeric antiparallel G4 formed by d(G4C2)_4_. The K^+^ ions are shown in purple spheres. O4′ oxygens are in yellow, and the cytosine bases are in orange. (**C**) Schematic representation of topology adopted by d(G4C2)_4_-anti. Arrows represent the 5′-to-3′ strand progression. The backbones of the core and loops are colored in red and green, respectively. W and N represent wide and narrow grooves. The guanine bases that adopt *syn* conformation are in hot pink, and those in the *anti* conformation are in cyan.

**Figure 5.**
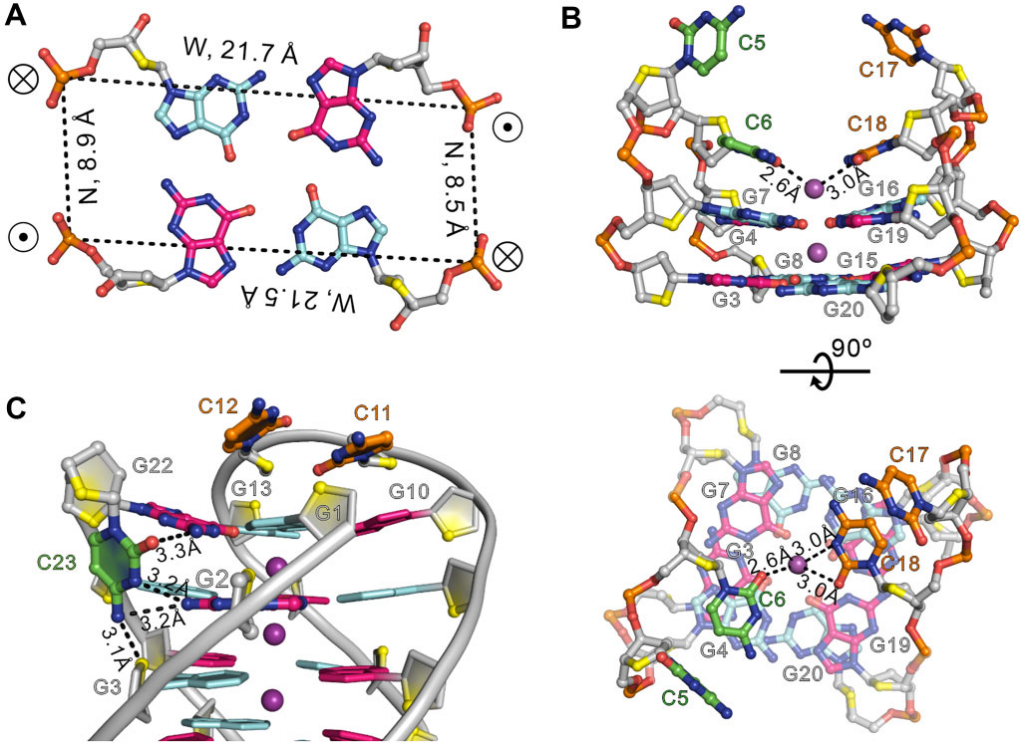
The detailed structure is d(G4C2)_4_-anti. (**A**) Illustrative example of medium groove formed by core bases G2·G9·G14·G21. Average groove width values are indicated by phosphate–phosphate distances shown as dashed lines. ⊗: 5′→3′ strand direction pointing into the paper. ⊙: 5′→3′ strand direction pointing toward the viewer. (**B**) The conformation of C5-C6 and C17-C18 in d(G4C2)_4_-anti. The C6 and C18 bases stack onto a G-tetrad layer, G4·G7·G16·G19, by a coordinating K^+^ ion. (**C**) The C23 are stabilized by the hydrogen bonds G2N2-C23N3, G2N2-C23N4, G3O4′-C23N4, and G22N2-C23O2, which are observed in chain A. The hydrogen bonds are shown in dashed lines. Phosphate backbone is in gray; phosphorus and phosphate oxygens are orange and red; and O4′ oxygens are in yellow. K^+^ ions are colored in purple.

The d(G4C2)_4_-anti was also successfully crystallized, and its structure was determined by molecular replacement using the NMR structure of d[(G4C2)3GG^Br^GG] (PDB: 2N2D) [15] as the searching model. The asymmetric unit contains four independent oligonucleotide chains, designated chains A through D (Supplementary Fig. S6), with well-resolved electron density for most nucleotides, except for the 3′-flanking bases C23, C24, and loop residues C11 and C12 (Figs 4A and B and Supplementary Fig. S7). Structural superposition of the four G4 units reveals a conserved G-core and CC loop architecture, while highlighting conformational variability at the 3′-end, particularly for C23 and C24, which adopt multiple orientations (Supplementary Fig. S7) (see below). The crystal structure reveals a monomeric antiparallel chair-type G4 composed of four stacked G-tetrads in which the hydrogen-bond directionalities are clockwise (G1→G10→G13→G22), anti-clockwise (G2←G9←G14←G21), clockwise (G3→G8→G15→G20), and anti-clockwise (G4←G7←G16←G19) patterns (Fig. 4C) with all Hoogsteen hydrogen bonds of N1-O6 and N2-N7 from the electron pair acceptor to the electron pair donor. Three centrally aligned K^+^ ions stabilize the G-core by coordinating with the guanine O6 atoms of the tetrads and an additional K^+^ ion is observed between bases C6, C18, and the G4·G7·G16·G19 tetrad layer (Fig. 4B). The glycosidic conformations of the guanines alternate in a *syn*·*anti*·*syn*·*anti* pattern, consistent with the antiparallel topology. The G-core is connected by three edgewise CC loops, resulting in two wide grooves with widths of 21.5/21.7 Å and two narrow grooves with widths of 8.5/8.9 Å (Figs 4C and 5A). The first and third loops containing C5, C6, C17, and C18 (at the bottom) span narrow grooves, while the second loop composed of C11 and C12 (on the top) spans a wide groove (Fig. 4C).

In the crystal structure of d(G4C2)_4_-anti, the C5 and C17 bases extend outward from the G-core (Fig. 5B and Supplementary Fig. S7). In contrast, the C6 and C18 bases stack onto the G4·G7·G16·G19 G-tetrad layer in coordination with a K^+^ ion forming an antiprismatic coordination environment with distances of 2.6 Å and 3.0 Å from the O2 atom of C6 and the O2 and N3 atoms of C18 (Fig. 5B). Interestingly, intermolecular interactions are also observed involving C5, C6, and C18, where the N4 atom of C5 from neighboring chain D′ forms two hydrogen bonds with the O2 atoms of C6 and C18 from chain A with distances of 2.9 Å, respectively, further stabilizing the local structure (Supplementary Fig. S8A).

The C11 base is present in chains A, B, and C but absent in chain D, while the C12 base is missing in chains C and D (Supplementary Fig. S7). The C11 and C12 bases are almost parallel to and stacked with the G1·G10·G13·G22 G-tetrad via π–π stacking interaction, except for the C11 and C12 in chain A, which tilt upward slightly (Fig. 5C and Supplementary Fig. S9). Notably, additional intermolecular interactions for C11 and C12 are observed, including the intermolecular π–π stacking of C11 and the intermolecular hydrogen bond C11N4-C12N3, which bridges the G4s formed by chains A and B in the asymmetric unit of crystal (Supplementary Fig. S8B).

Similarly, the C23 base is observed in chains A, C, and D but missing in chain B, while the C24 base is absent in chains C and D (Supplementary Fig. S7). In chain A, despite the absence of C24, C23 forms a robust hydrogen-bonding network involving the hydrogen bonds G2N2-C23N3, G2N2-C23N4, G3O4′-C23N4, and G22N2-C23O2. These interactions insert C23 into the wide groove (Fig. 5C). In Chain C, where C11 and C12 are missing, the C23 and C24 bases adopt a near-parallel orientation and stack against the G1·G10·G13·G22 G-tetrad (Supplementary Fig. S9B). Interestingly, in chain D, the C23 and C24 are oriented oppositely, with the C24 base pointing outward but bridged to C11 via hydrogen bonds C11N3-C24O3′ and C11N4-C24O2 (Supplementary Fig. S9C).

### Comparison of d(G4C2)_4_-anti with other G4s formed by G4C2 repeats

Previous studies have reported two monomeric antiparallel G4 structures formed by d[(G4C2)_3_G4] determined using NMR method under different conditions: the NAN structure (PDB: 2N2D), resolved under neutral pH with annealing at pH 7.2 [15], and the AQU structure (PDB: 5OPH), resolved under acidic and quenching conditions at pH 5.8 [18]. Both structures incorporate an 8Br-dG residue at position 21 to facilitate structure determination. The crystal structure of d(G4C2)_4_-anti was solved by molecular replacement using the NAN structure as a search model. Structural alignment revealed that d(G4C2)_4_-anti is similar to NAN, with an RMSD of ∼0.722 Å, indicating a conserved architecture composed of four G-tetrad layers and three lateral loops (Supplementary Fig. S10A). In both d(G4C2)_4_-anti and NAN structures, the C11 and C12 bases are oriented nearly parallel to the neighboring G-tetrad layer, except for C11 of d(G4C2)_4_-anti, which is tilted ∼45° (Supplementary Fig. S10A). Likewise, the C6 and C18 bases in the C5-C6 and C17-C18 loops are also stacked parallel to the adjacent G-tetrad layer in both structures. However, a notable difference lies in the positions of C5 and C17: in d(G4C2)_4_-anti, these bases extend outward from C6 and C18, whereas in NAN, C5 and C17 are situated directly above C6 and C18 (Supplementary Figs S10A and S10B). Importantly, d(G4C2)_4_-anti features an additional K^+^ ion that bridges the C6 and C18 bases with the G4·G7·G16·G19 layer (Figs. 5B and 5C). Although K^+^ ions were not detected by NMR in the NAN structure, the similarity of C5 and C17 in both structures suggests that a corresponding K^+^ ion may exist in NAN but remain undetected under NMR conditions.

On the other hand, ANU also forms a monomeric antiparallel G4 with four G-tetrad layers and three lateral loops, but differs from NAN and d(G4C2)_4_-anti in the directionality of Hoogsteen hydrogen bond within each G-tetrad [18]. In AQU, the C12 base is parallel to the adjacent G-tetrad layer, as observed in both NAN and d(G4C2)_4_-anti structures, while C11 is tilted ∼45° relative to the adjacent G-tetrad layer and positioned above C12 (Supplementary Fig. S10C). Intriguingly, AQU contains two independent C·C base pairs, comprising C5N3-C18N4, C6N3-C17N4, C5N4-C17N3, and C6N4-C17N3 hydrogen bonds, that are parallel to and stacked with the neighboring G-tetrad layer (Supplementary Fig. S10C). By contrast, such C·C base pairs are absent in both NAN and d(G4C2)_4_-anti, potentially because the C5-C6 and C17-C18 edge loops span the wide groove in AQU but the narrow groove in NAN and d(G4C2)_4_-anti (Fig. 4 and Supplementary Fig. S10C).

### Structural diversity of G4C2 G4s

To further evaluate this structural diversity of G4C2 G4s, we systematically analyzed the backbone dihedral angles (α, β, γ, δ, ϵ, ζ, describing the nucleic acid backbone), glycosidic torsion angles (χ), and sugar puckering conformations of current available G4C2 G4 structures. These include antiparallel-folded G4s (i.e. d(G4C2)_4_-anti, NAN [15], and AQU [18]) (Supplementary Fig. S11) and parallel-folded G4s (i.e. d(G4C2)_4_-para, d(G4C2)_2_ [20], and r(G4C2)_2_ [21]) (Supplementary Fig. S12). In terms of sugar pucker, most bases in DNA G4s formed by *C9orf72* G4C2 repeats favor a C2′-*endo*/C1′-*exo* conformation, whereas approximately half of the bases in RNA adopt a C3′-*endo* conformation. Notably, several bases in both DNA and RNA G4s exhibit an O1′-*endo* conformation, underscoring local structural variability (Supplementary Table S1).

Backbone torsion angle analysis revealed considerable flexibility across the α, β, and γ angles, reflecting the intrinsic adaptability of nucleic acid backbones under different topological constraints. The δ angles are more ordered yet remain variable, while ϵ angles fluctuate with distinct patterns depending on the topology. In antiparallel structures, AQU displays more pronounced ϵ angle fluctuations compared to d(G4C2)_4_-anti and NAN, possibly due to the reversed Hoogsteen hydrogen bonding directionality in its G-tetrads. In contrast, ϵ angles in parallel structures tend to fluctuate in a more ordered manner, with lower amplitude, although d(G4C2)_4_-para is an exception. Specifically, d(G4C2)_4_-para shows larger-amplitude ϵ angle fluctuations at G3, G10, G15, G20, and G22, contributing to the difference in groove width of ∼0.6 Å. In contrast, the significantly fluctuating ϵ angles of G1, G10, G13, G19, and G21 in d(G4C2)_4_-anti result in a narrower major groove and a wider minor groove. Comparable ϵ angle fluctuations are also observed in the NAN and AQU structures (Supplementary Table S2). The ζ angles in parallel G4s exhibit more pronounced fluctuations than those in antiparallel ones, reflecting the sensitivity of this torsion angle to specific folding architectures. χ angle analysis further highlights conformational diversity within loop regions, with select cytosines—such as C12 in d(G4C2)_4_-anti and C11/C17 in d(G4C2)_4_-para—adopting the *syn* conformation (Supplementary Figs S11 and S12). These local variations may significantly impact base accessibility and recognition by interacting proteins or small molecules.

Taken together, these findings highlight the pronounced conformational flexibility of G4s formed by G4C2 repeats, particularly in the DNA context. The variations in torsion angles and sugar pucker conformations not only facilitate diverse G4 topologies but may also modulate interactions with cellular factors, potentially influencing their stability and pathogenicity. The distinct divergence in backbone conformation and sugar puckering between DNA and RNA G4s highlights their unique structural adaptabilities, potentially contributing to their differential stabilities and unique roles in *C9orf72*-related pathogenesis.

## Discussion

G-rich nucleic acid sequences can fold into G4s with diverse topologies. This structural polymorphism is influenced by multiple factors, including sequence length and content, the presence and identity of monovalent cations, concentration, ionic strength, and the pH [31–33]. Both experimental and computational studies have uncovered intricate folding pathways involving transient intermediate states, such as G-triplexes and hairpins, which act as key precursors to fully formed G4 folding topologies [34–37]. In particular, the nature of flanking sequences and the loop residues has been shown to significantly influence the topological equilibrium among different G4 conformations [38, 39].

In the context of *C9orf72*-associated ALS/FTD, the G4C2 hexanucleotide repeat has been extensively studied due to its propensity to form polymorphic G4 structures [14, 15, 17, 18, 20, 21, 40]. Previous research has demonstrated that both repeat length and environmental conditions—particularly pH—play pivotal roles in determining the topology of G4s formed by G4C2 sequences. Our present work extends this understanding by elucidating the crystal structures of two distinct conformations formed by the same d(G4C2)_4_ sequence: a parallel-stranded dimer (d(G4C2)_4_-para) and an antiparallel monomer (d(G4C2)_4_-anti). The unique structural features we identify, such as propeller versus edgewise loop arrangements, 5′–5′ π–π stacking, and base stacking interactions involving terminal cytosines, likely mediate the transition from dynamic folding intermediates into specific stable architectures.

To place our findings in the broader context of known G4 structural diversity, we performed a systematic search of the Protein Data Bank (PDB; accessed 9 July 2025), which revealed 446 G4 structures determined by NMR or X-ray crystallography. Of these, only 22 unimolecular G4 structures derived from 18 unique nucleotide sequences and excluding *C9orf72*-derived G4C2 repeats, contain four stacked G-tetrads, highlighting the rarity and biological significance of such structures (Supplementary Table S3).

Among these 22 cases, only one is an RNA sequence, r[(GGA)_4_A(GGA)_4_], an RNA aptamer that blocks pathological conformational conversion of prion protein. This aptamer forms a parallel four-layered G4, in which the central two G-tetrads form two G·A·G·G·A·G hexads that stack onto each other via π–π interactions [41]. In contrast, five DNA sequences form antiparallel four-layered G4s with a basket-type topology. These include two classical *Oxytricha* telomeric sequences, d[G4(T4G4)_3_] [42] and its uracil- and inosine-containing derivative d(G4TUTUG4T4G4UUTTG3I) [43], two DNA sequences d(G4T*n*G4T4G4A2G4) (where *n*= 2 or 3) [44], and one DNA sequence, d(G4GAG4TACAG4TACAG4), from *Dictyostelium discoideum* genome [45]. These antiparallel G4s are characterized by *syn·syn·anti·anti* glycosidic torsion angles in each G-tetrad and are connected via two lateral loops and one diagonal loop. A unique four-layered G4 formed by the DNA sequence *AT11*, d[T(GGT)_3_TGTTG(TGG)_3_TGGT], derived from the anti-proliferative aptamer *AGRO100* (also known as *AS1411*), consists of two parallel-stranded subunits connected by a central T residue [46]. These subunits stack through 3′–5′ end interactions, generating a stable, pseudo-unimolecular parallel G4 structure. Furthermore, a variant of the *Tetrahymena thermophila* telomeric DNA repeat (TTGGGG)_n_, d(GTTG4TTG4GTTG4TTG4T), known as TET26, was reported to form a parallel four-tetrad unimolecular G4 [47]. Intriguingly, a variant of TET26, d(GTTG4TTG4GTTG4TTG4), known as TET25, forms a (3 + 1) hybrid four-layer G4 with a 5′ snapback and three lateral loops plus one propeller loop. This structure exhibits mixed glycosidic conformations: *syn·anti·anti·anti* in three tetrads and *syn·syn·anti·anti* in one [47].

Interestingly, 10 of the 18 unimolecular G4s adopted left-handed or right-left hybrid parallel conformations. The left-handed *Z-G4* formed by d[T(GGT)_3_GGTTG(TGG)_3_TGTT] contains two parallel two-layered G4 units stacking onto each other [48]. Two minimal left-handed G4 motifs, d[GT(GGT)_3_G] and d[(GGT)_3_GTG], were identified as building blocks of Z-G4s [49, 50]. When joined via two T residues as d[GT(GGT)_3_GTT(GGT)_3_GTG], these motifs form a right-left hybrid parallel G4 structure (PDB: 9CIY). Interestingly, the thrombin-binding aptamer fused to the minimal left-handed G4 motif d[G2T2G2TGTG2T2G2T*n*GT(GGT)_3_G] (where *n*= 1 or 2), can fold into a right-left hybrid G4 with parallel topology [51]. Collectively, these examples emphasize that four-layered G4s are structurally rare, heterogeneous, and may play specialized biological roles.

Our structural analysis of d(G4C2)_4_ underscores the conformational diversity of G4C2 G4s. We demonstrate that the same d(G4C2)_4_ sequence can adopt both monomeric and dimeric G4s under similar conditions, emphasizing the structural plasticity of these repeats. The biological significance of this oligomerization remains unknown but is potentially relevant to ALS/FTD pathogenesis. In structural terms, the d(G4C2)_4_-para folds into an eight-layered G4 stabilized by 5′-5′ π–π stacking, a central K^+^ ion, and an intermolecular C·C^+^·C·C^+^ quadruple base pair. These distinct structural features may promote higher-order oligomerization of G4, potentially disrupting RNA polymerase activity or inducing genomic instability. In contrast, the d(G4C2)_4_-anti features edgewise loops and an additional K^+^ ion mediating π-stacking between cytosines of edgewise loops and G-tetrads, which may alter chromatin structure or facilitate repeat expansion via slipped-strand mispairing. Thus, the oligomerization propensity of d(G4C2)_4_-para may drive toxic aggregation, while the dynamic topology of d(G4C2)_4_-anti may influence local nucleic acid dynamics. Nevertheless, these two proposed mechanisms might exhibit unequal contributions to disease pathogenesis, which requires systematic investigation in future studies.

Lastly, the unique conformational features identified here—including diverse backbone geometries, sugar puckering profiles, and groove dimensions—may serve as molecular recognition elements for the development of selective small molecules targeting *C9orf72* DNA G4s. The conformation-specific probes will enable direct visualization and validation of these distinct G4 topologies in disease-relevant cellular models, bridging our structural insights to pathological mechanisms. Furthermore, the well-defined and rigid nature of d(G4C2)_4_-para makes it an attractive target for small-molecule G4 stabilizers, potentially capable of interfering with toxic G4 aggregation in *C9orf72*-related ALS/FTD. In contrast, the dynamic topology and less defined binding surfaces of d(G4C2)_4_-anti complicate its therapeutic targeting. Structure-based ligand design efforts may thus preferentially focus on disrupting d(G4C2)_n_ oligomerization by targeting the parallel G4 topology as a strategy to interfere with *C9orf72*-driven disease processes.

## Supplementary Material

gkaf879_Supplemental_File

## Data Availability

Atomic coordinates and structure factors for the reported crystal structures of d(G4C2)_4_-para and d(G4C2)_4_-anti have been deposited with the Protein Data Bank under accession numbers 9UK6 and 9UK8, respectively.
