# Supplementary material for: Crystal structures of distinct parallel and antiparallel DNA G-quadruplexes reveal structural polymorphism in C9orf72 G4C2 repeats
Source: Nucleic Acids Res. 2025 Sep 10;53(17):gkaf879. doi: 10.1093/nar/gkaf879 (PMC12421379; doi:10.1093/nar/gkaf879)
Supplement: gkaf879_Supplemental_File [file gkaf879_supplemental_file.pdf]

Supporting Information

Crystal Structures of Distinct Parallel and Antiparallel DNA G-Quadruplexes Reveal Structural Polymorphism in C9orf72 G4C2 Repeats

Yanyan Geng[1,†,*], Changdong Liu[2,3,†], Haitao Miao[2], Monica Ching Suen[2], Yuanyuan Xie[4], Bingchang Zhang[4], Wanhong Han[4], Caiming Wu[5] , Haixia Ren[5], Xueqin Chen[1], Hwan-Ching Tai[6], Zhanxiang Wang[4,*] Guang Zhu[2,3,*],and Qixu Cai[6,*]

[1] Clinical Research Institute of the First Affiliated Hospital of Xiamen University, Fujian Key Laboratory of Brain Tumors Diagnosis and Precision Treatment, Xiamen Key Laboratory of Brain Center, the First Affiliated Hospital of Xiamen University, School of Public Health, School of Medicine, Xiamen University, Xiamen 361003, Fujian, China

[2] Institute for Advanced Study and State Key Laboratory of Molecular Neuroscience, Division of Life Science, The Hong Kong University of Science and Technology, Clear Water Bay, Kowloon, Hong Kong SAR, China

[3] HKUST Shenzhen Research Institute, Hi-Tech Park, Nanshan, Shenzhen 518057, Guangdong, China

[4] Department of Neurosurgery and Department of Neuroscience, Fujian Key Laboratory of Brain Tumors Diagnosis and Precision Treatment, Xiamen Key Laboratory of Brain Center, the First Affiliated Hospital of Xiamen University, School of Medicine, Xiamen University, Xiamen 361003, Fujian, China

[5] State Key Laboratory of Cellular Stress Biology, School of Life Sciences, Xiamen University, Xiamen 361105, Fujian, China

[6] State Key Laboratory of Vaccines for Infectious Diseases, School of Public Health, Xiamen University, Xiamen 361105, Fujian, China

* To whom correspondence should be addressed. Email: qxcai@xmu.edu.cn; gzhu@ust.hk; wangzx@xmu.edu.cn; yygeng@xmu.edu.cn

[†] The authors wish it to be known that, in their opinion, the first 2 authors should be regarded as joint First Authors.

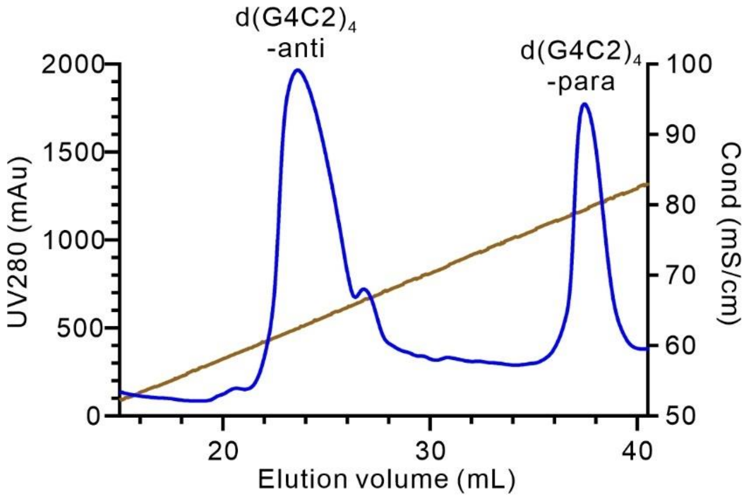

Figure S1. The anion exchange chromatography of d(G4C2)$_4$ showing two separated fractions. The binding buffer contains 70 mM KCl and 20 mM potassium phosphate (pH 7.0), while the elution buffer consists of 1 M KCl and 20 mM potassium phosphate (pH 7.0). The two main components, d(G4C2)$_4$-anti and d(G4C2)$_4$-para were completely separated by a linear gradient elution with a range of 500 mM to 900 mM KCl.

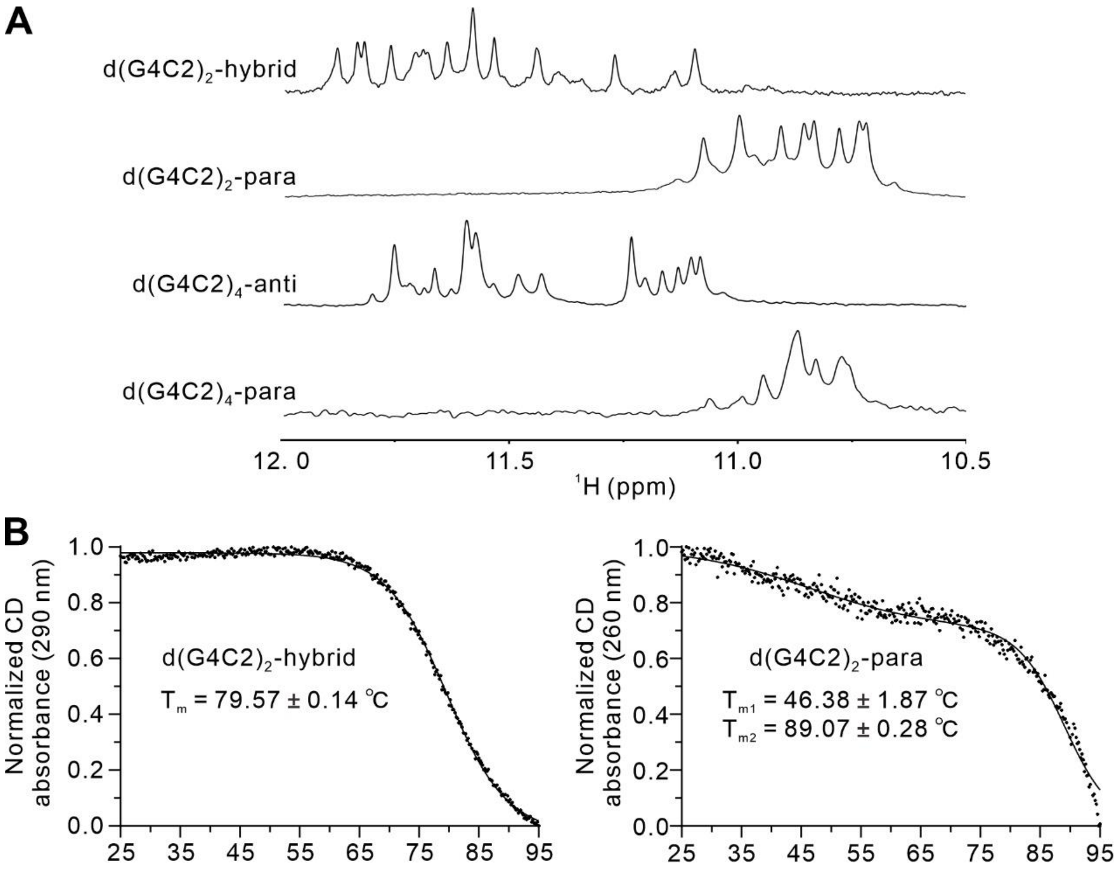

Figure S2. Comparison with G4s formed by d(G4C2)$_2$. (A) The imino region of 1D ^{1}H-NMR spectra of d(G4C2)$_2$-hybrid and d(G4C2)$_2$-para in 20 mM potassium phosphate solution containing 70 mM KCl with pH 7.0 recorded at 25 °C on 850 MHz. (B) CD melting curves of d(G4C2)$_2$-hybrid and d(G4C2)$_2$-para in 20 mM potassium phosphate solution containing 70 mM KCl with pH 7.0. Data were fitted by the Boltzmann sigmoid equation or double sigmoidal equation (GraphPad Prism).

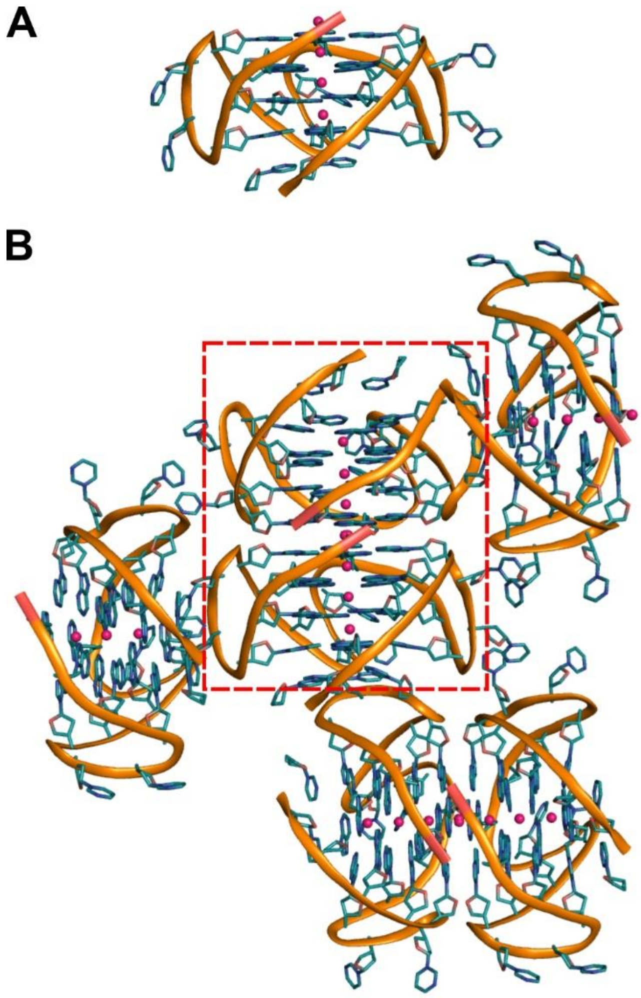

Figure S3. The crystal structure of d(G4C2)$_4$-para in I4$_1$ space group. (A) There is only one oligonucleotide strand of d(G4C2)$_4$ in an asymmetric unit of crystal. (B) Crystal packing of d(G4C2)$_4$-papra. The dimeric G4 is indicated by dashed red rectangles.

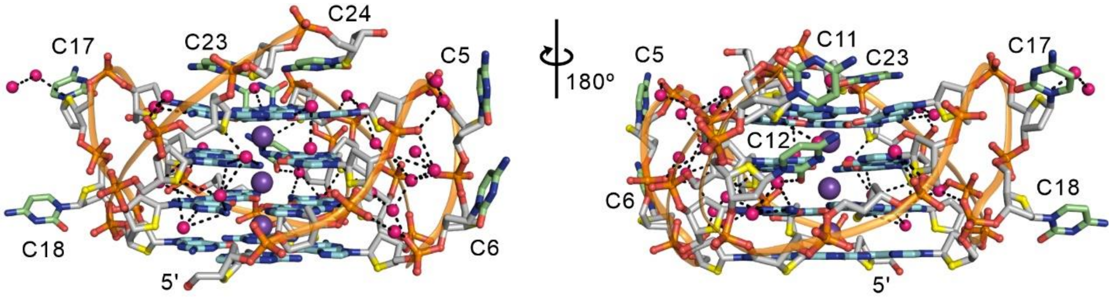

Figure S4. Water clusters and networks in d(G4C2)$_4$-para. Water networks are depicted in all four medium grooves. O4' oxygens are in yellow, the cytosine bases are in green. The expanded region of water-mediated C11-C12 conformation in right are shown in Figure 3E. All the hydrogen bonds are represented by dash lines. The water molecules are shown in red sphere and K+ ions are colored by magenta.

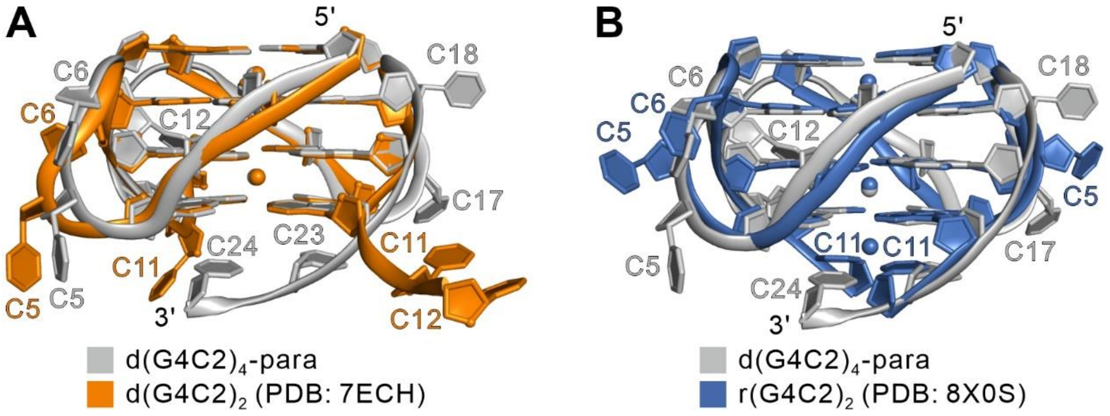

Figure S5. The structural comparison of d(G4C2)-para (grey) with (A) d(G4C2)$_2$ (orange, PDB: 7ECH) and (B) r(G4C2)$_2$ (blue, PDB: 8X0S). The K^+ ions are shown in sphere.

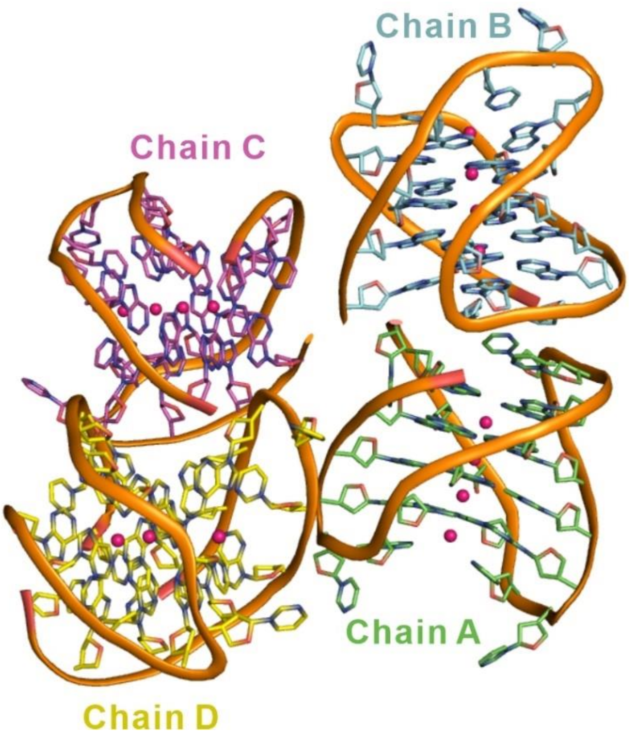

Figure S6. The crystal structure of d(G4C2)$_4$-anti contains four chains in an asymmetric unit.

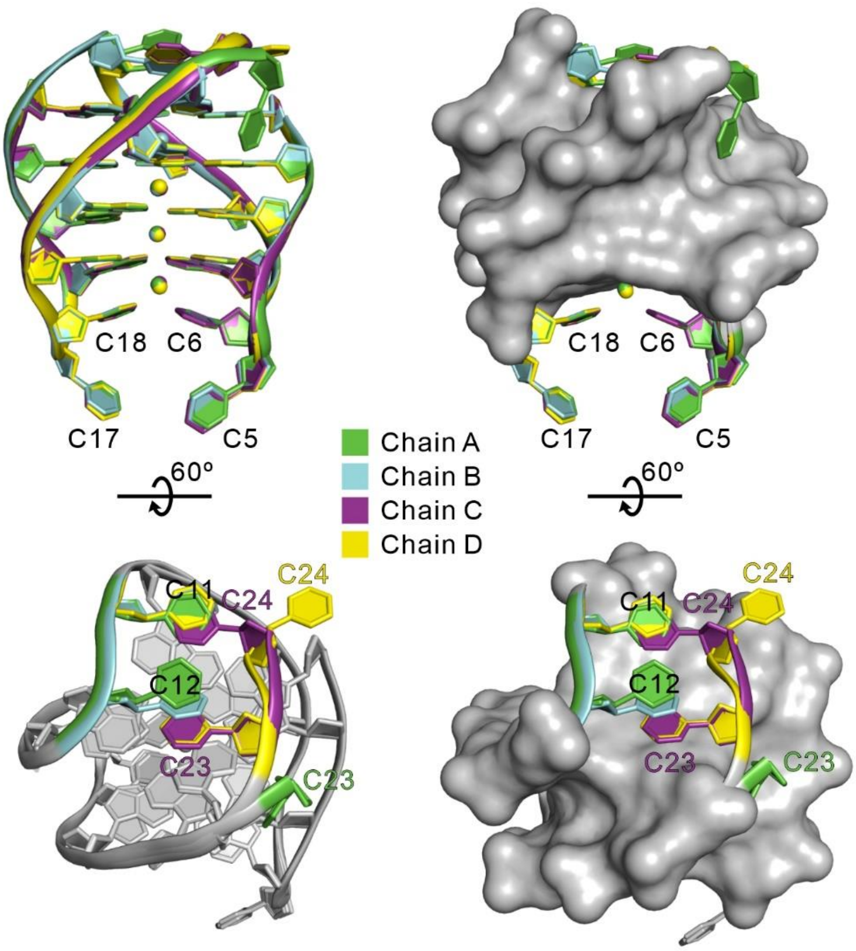

Figure S7. The superposed G4 structures formed by chain A (green), chain B (cyan), chain C (purple) and chain D (yellow). The all-heavy-atom root-mean-square deviation (RMSD) values of chain B, C and D superposed with chain A are ~0.052, ~0.060 and ~0.074 Å. The C24 is missing in chain A. The C23 and C24 are missing in chain B. The C11 and C12 are missing in chain C. The C12 is missing in chain D.

	chain A (green)	chain B (cyan)	chain C (purple)	chain D (yellow)
C11	✔	✔	✕	✔
C12	✔	✔	✕	✕
C23	✔	✕	✔	✔
C24	✕	✕	✔	✔

✔ : observed; ✕ :missing.

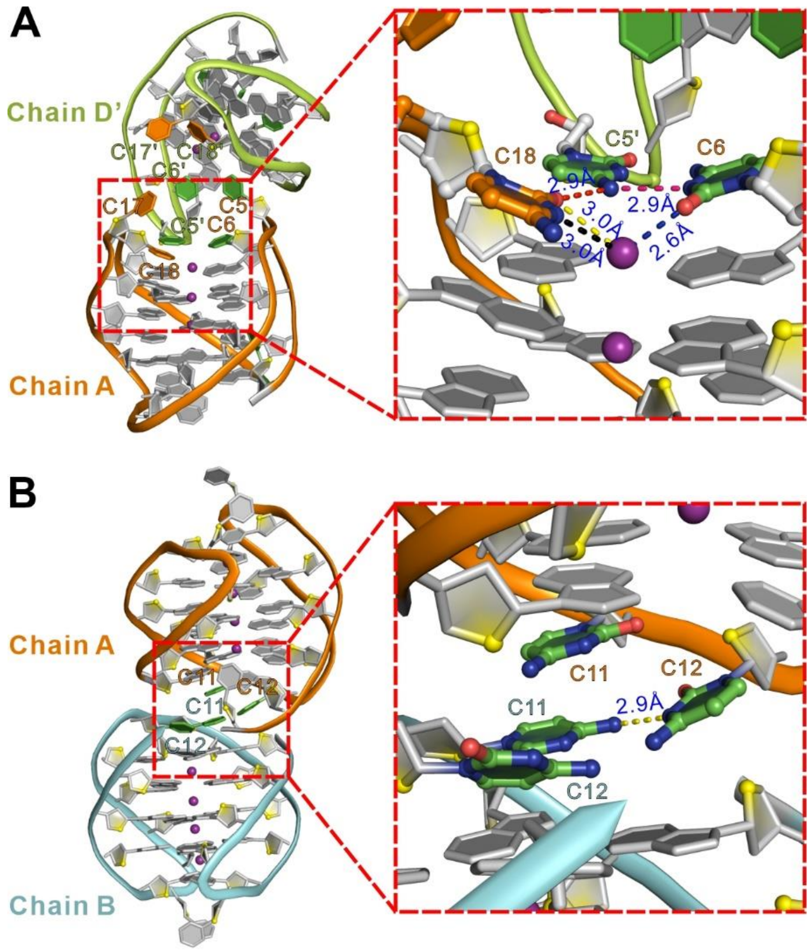

Figure S8. The intermolecular interaction of cytosine bases in d(G4C2)$_4$-anti. (A) The intermolecular interaction of C5, C6 and C18 in d(G4C2)$_4$-anti. (B) The intermolecular interaction of C11 and C12 in d(G4C2)$_4$-anti. The hydrogen bonds are shown in dashed yellow lines.

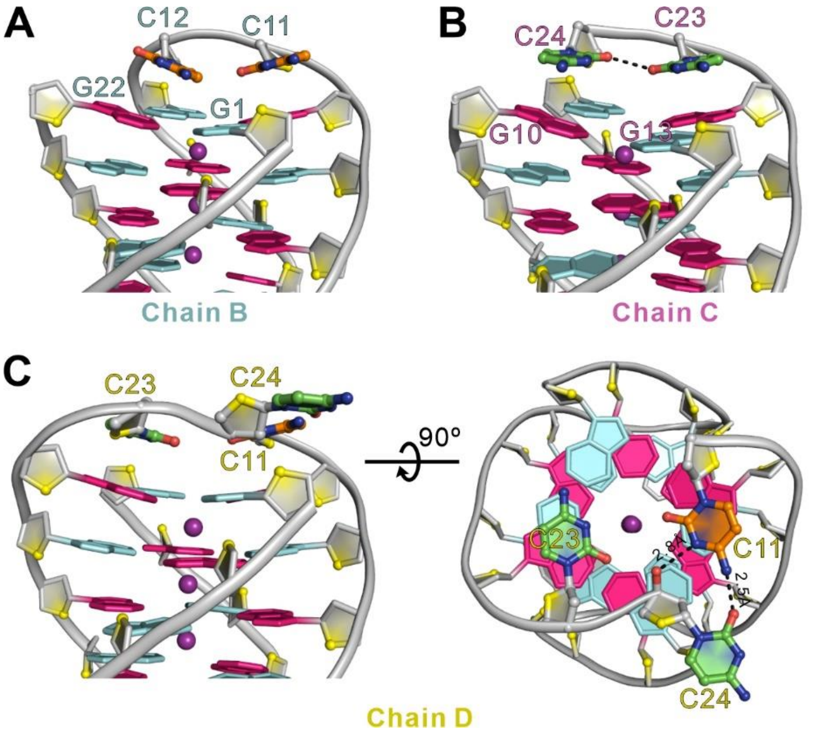

Figure S9. The conformation of C11, C12, C23 and C24 in the chain B (A), C (B) and D (C) of d(G4C2)$_4$-anti. The hydrogen bonds are shown in dashed lines. The K^+ ions are shown in purple sphere. The C11 and C12 are in orange. The C23 and C24 are in green. The guanine bases of G-terads adopt *syn* conformation are in hotpink and *anti* conformation are in cyan. O4' oxygens are in yellow.

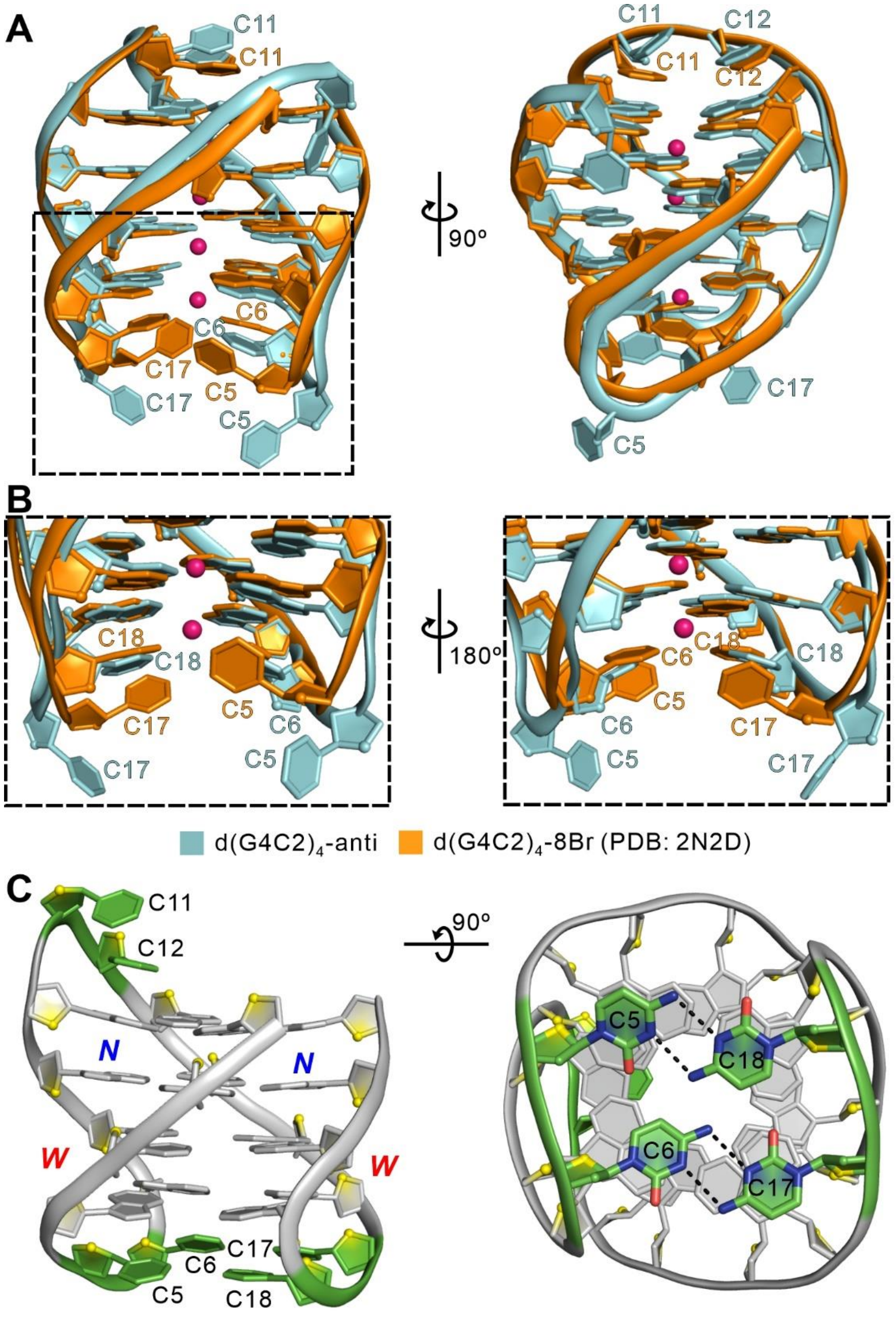

Figure S10. The structural comparison of d(G4C2)-anti (cyan) with NAN (PDB:2N2D, orange) and AQU (PDB:5OPH). (A-B) The superimposition (A) of d(G4C2)-anti and NAN, which are shown in cartoon and the expanded region (B) for the conformation of C5, C6 and C17, C18. The K^+ ions are colored hotpink in sphere. (C) The representative structure of AQU in cartoon mode (left) and the two C・C base pairs (Right). The cytosine bases are colored green and the O4' atoms are colored yellow. W and N indicate wide and narrow groove, respectively.

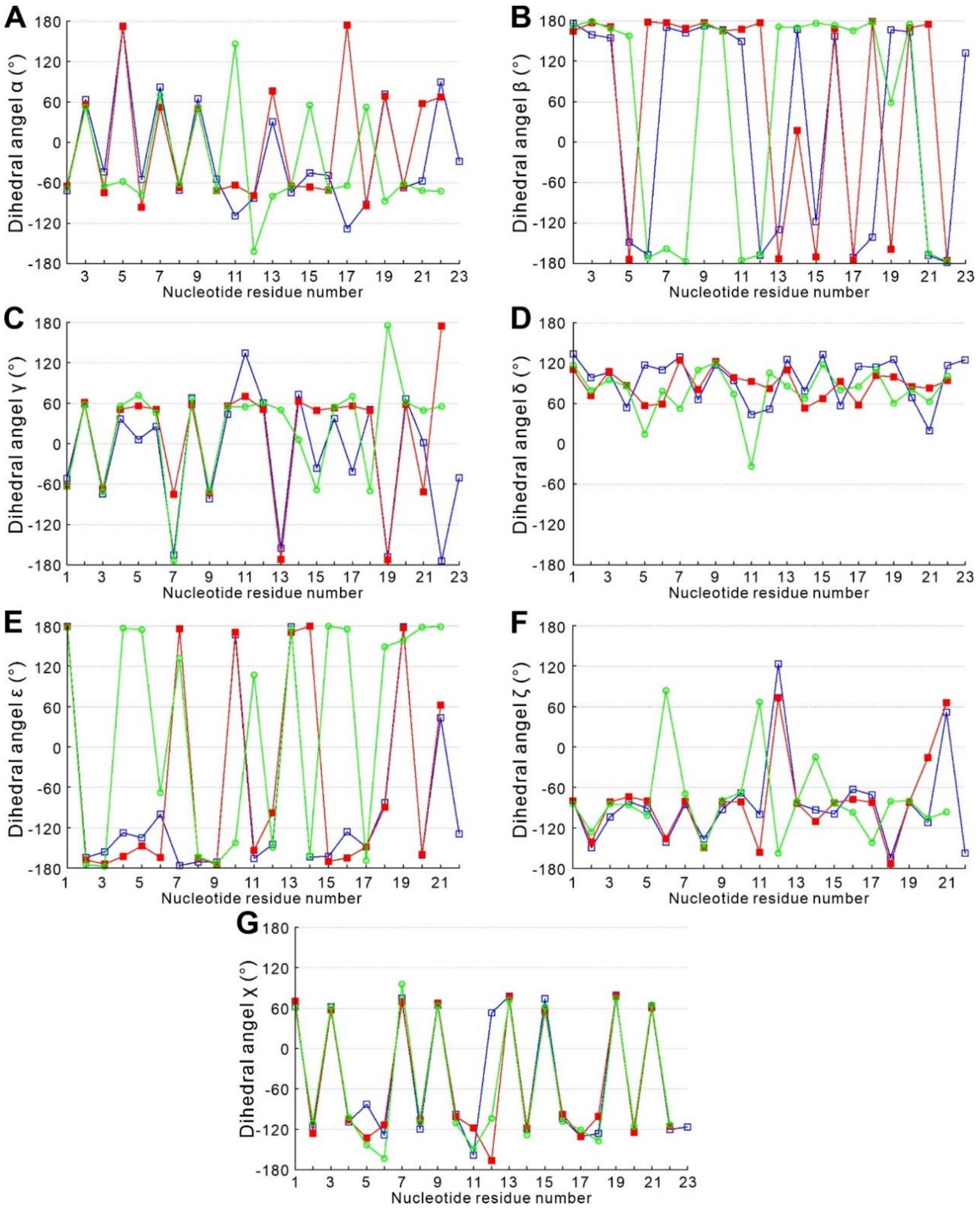

Figure S11. The distribution of dihedral angles in d(G4C2)$_4$-anti, NAN (PDB:2N2D), and AQU (PDB:5OPH), respectively. The d(G4C2)$_4$-anti (blue), NAN (red) and AQU (yellow) fold into antiparallel G4s. Six dihedral angles, (A) α, (B) β, (C) γ, (D) δ, (E) ε and (F) ζ describe DNA backbone; dihedral angle (G) χ refers to *syn* vs *anti* nucleobase conformation.

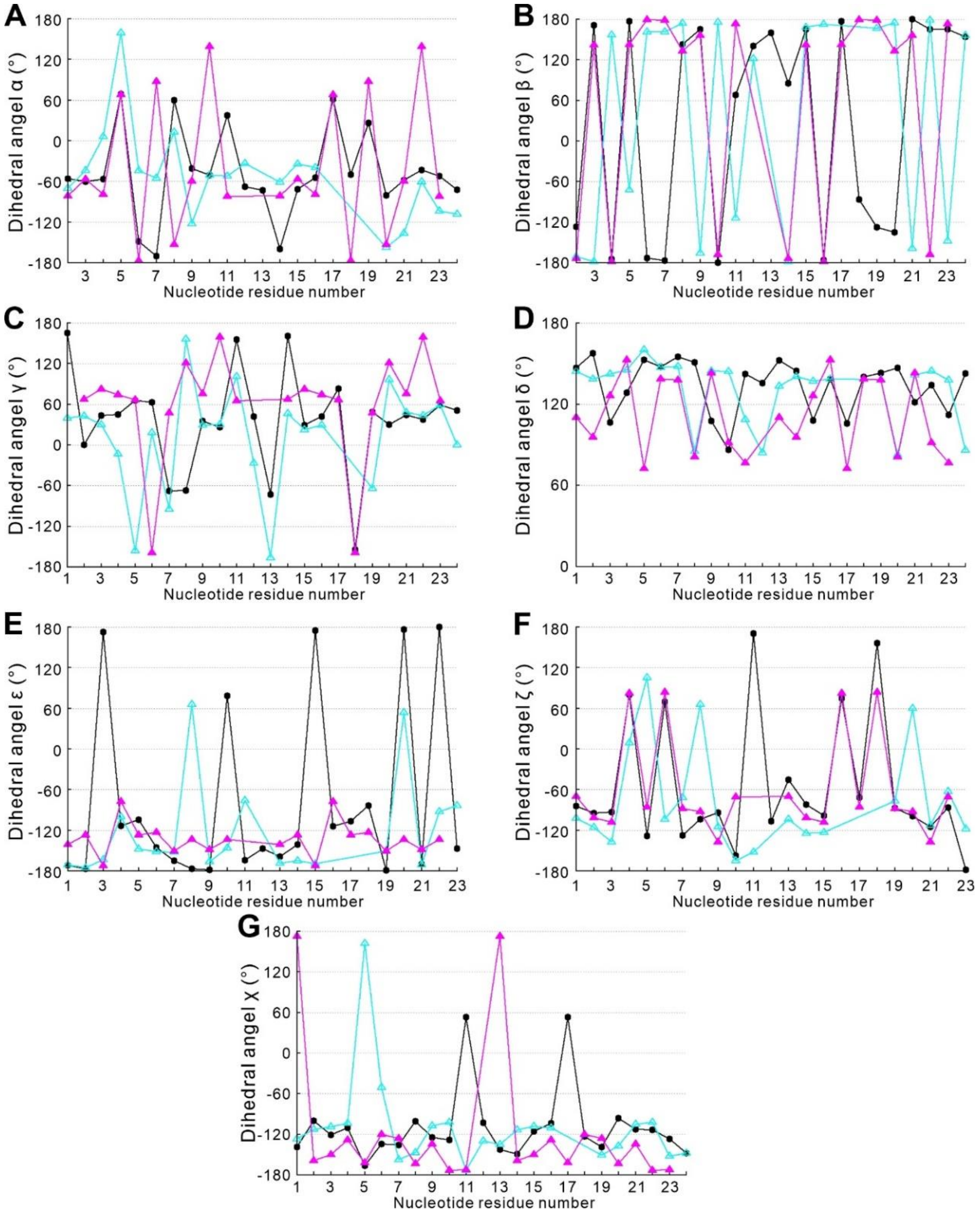

Figure S12. The distribution of dihedral angles in d(G4C2)$_4$-para, d(G4C2)$_2$ (PDB: 7ECH) and r(G4C2)$_2$ (PDB: 8X0S). The d(G4C2)$_4$-para (black), d(G4C2)$_2$ (cyan) and r(G4C2)$_2$ (magenta) fold into parallel G4s. Six dihedral angles, (A) α, (B) β, (C) γ, (D) δ, (E) ε and (F) ζ describe DNA/RNA backbone; dihedral angle (G) χ refers to *syn* vs *anti* nucleobase conformation.

Table S1. Sugar pucker conformations of d(G4C2)-anti, NAN (PDB:2N2D), AQU (PDB:5OPH), d(G4C2)-para, $d(G4C2)_2$ (PDB: 7ECH) and $r(G4C2)_2$ (PDB: 8X0S). The analysis was done by Curve+ (1). The *C1'-exo* sugar pucker is in green; *C2'-endo* sugar pucker is in red; *C3'-exo* sugar pucker is in lemon; *C3'-endo* sugar pucker is in blue; *C4'-exo* sugar pucker is in cyan; *O1'-endo* sugar pucker is in purple. The bases shown as black dashes indicate that bases are missing or not involved in PDB.

	d(G4C2)-anti	NAN	AQU	d(G4C2)-para	$d(G4C2)_2$	$r(G4C2)_2$
**G1**	*C2'-endo*	*C2'-endo*	*C2'-endo*	*C2'-endo*	*C2'-endo*	*C2'-exo*
**G2**	*C2'-endo*	*C1'-exo*	*C1'-exo*	*C3'-exo*	*C2'-endo*	*C3'-endo*
**G3**	*C2'-endo*	*C2'-endo*	*C2'-endo*	*O1'-endo*	*C2'-endo*	*C1'-exo*
**G4**	*C1'-exo*	*C1'-exo*	*C2'-endo*	*C1'-exo*	*C2'-endo*	*C2'-endo*
**C5**	*C2'-endo*	*C1'-exo*	*O1'-endo.*	*C3'-exo*	*C4'-endo*	*C3'-endo*
**C6**	*C3'-exo*	*C1'-exo*	*C1'-exo*	*C2'-endo*	*C3'-exo*	*C2'-endo*
**G7**	*C3'-exo*	*C2'-endo*	*C1'-exo*	*C2'-endo*	*C2'-endo*	*C2'-endo*
**G8**	*C1'-exo*	*C1'-exo*	*C2'-endo*	*C3'-exo*	*C4'-exo*	*C4'-exo*
**G9**	*C2'-endo*	*C2'-endo*	*C2'-endo*	*O1'-endo*	*C2'-endo*	*C2'-endo*
**G10**	*C2'-endo*	*C2'-endo*	*C1'-exo*	*C4'-exo*	*C2'-endo*	*C3'-endo*
**C11**	*C1'-exo*	*C2'-endo*	*C3'-endo*	*C2'-endo*	*O1'-endo*	*C3'-endo*
**C12**	*C1'-exo*	*C1'-exo*	*C2'-endo*	*C2'-endo*	*C4'-exo*	----
**G13**	*C2'-endo*	*C2'-endo*	*C2'-endo*	*C2'-endo*	*C2'-endo*	*C2'-exo*
**G14**	*C1'-exo*	*C1'-exo*	*C1'-exo*	*C2'-endo*	*C2'-endo*	*C3'-endo*
**G15**	*C2'-endo*	*C1'-exo*	*C2'-endo*	*C1'-exo*	*C2'-endo*	*C1'-exo*
**G16**	*C1'-exo*	*C2'-endo*	*C2'-endo*	*C2'-endo*	*C2'-endo*	*C2'-endo*
**C17**	*C2'-endo*	*C1'-exo*	*C2'-endo*	*O1'-endo*	----	*C3'-endo*
**C18**	*C2'-endo*	*C2'-endo*	*C2'-endo*	*C2'-endo*	----	*C2'-endo*
**G19**	*C3'-exo*	*C2'-endo*	*C1'-exo*	*C2'-endo*	*C2'-endo*	*C2'-endo*
**G20**	*C1'-exo*	*C1'-exo*	*C2'-endo*	*C2'-endo*	*O1'-endo*	*C4'-exo*
**G21**	*O1'-endo*	*C2'-endo*	*C1'-exo*	*C1'-exo*	*C2'-endo*	*C2'-endo*
**G22**	*C2'-endo*	*C2'-endo*	*C2'-endo*	*C2'-endo*	*C2'-endo*	*C3'-endo*
**G23**	*C3'-exo*	----	----	*C1'-exo*	*C2'-endo*	*C3'-endo*
**G24**	----	----	----	*C2'-endo*	*C3'-endo*	----

Table S2. The width of four grooves observed in structure of d(G4C2)$_4$-para, d(G4C2)$_4$-anti, NAN and AQU. M1, M2, M3 and M4 indicates medium groove 1 between G1 and G7, medium groove 2 between G7 and G13, medium groove 3 between G13 and G19 and medium groove 4 between G19 and G1 in G1·G7·G13·G19 layer of d(G4C2)$_4$-para. N1, W2, N3 and W4 indicates narrow groove 1 between G1 and G10, wide groove 2 between G10 and G13, narrow groove 3 between G13 and G21 and wide groove 4 between G21 and G1 in G1·G10·G13·G21 layer of d(G4C2)$_4$-anti and NAN, whereas W1, N2, W3 and N4 in AQU. Similarly, for the other layers. The average groove width values are indicated by phosphate-phosphate distances in each G-tetrad. The underlined bases in bold display ε angles with large-amplitude fluctuations.

	**d(G4C2)$_4$-para**			
	**M1**	**M2**	**M3**	**M4**
G1 • G7 • G13 • G19*	15.0Å	15.0Å	15.4Å	15.4Å
G2 • G8 • G14 • **G20**	16.0Å	16.3Å	16.1Å	15.8Å
**G3** • G9 • **G15** • G21	16.5Å	17.7Å	16.5Å	16.8Å
G4 • **G10** • G16 • **G22**	16.4Å	16.7Å	16.6Å	16.3Å
	**d(G4C2)$_4$-anti**			
	**N1**	**W2**	**N3**	**W4**
**G1** • **G10** • **G13** • G22*	10.8Å	16.6Å	9.5Å	16.9Å
G2 • G9 • G14 • **G21**	8.5Å	21.7Å	8.9Å	21.5Å
G3 • G8 • G15 • G20	9.2Å	21.5Å	9.3Å	21.6Å
G4 • G7 • G16 • **G19**	9.9Å	19.0Å	9.9Å	19.4Å
	**NAN**			
	**N1**	**W2**	**N3**	**W4**
**G1** • **G10** • **G13** • G22*	11.8Å	16.6Å	11.8Å	17.9Å
G2 • G9 • **G14** • **G21**	9.4Å	21.6Å	8.7Å	22.1Å
G3 • G8 • G15 • G20	9.6Å	21.3Å	9.2Å	22.4Å
G4 • **G7** • G16 • **G19**	9.5Å	20.8Å	11.1Å	20.6Å
	**AQU**			
	**W1**	**N2**	**W3**	**N4**
**G1** • G10 • **G13** • G22*	17.4Å	12.6Å	18.0Å	10.9Å
G2 • G9 • G14 • **G21**	21.6Å	9.0Å	22.1Å	8.4Å
G3 • G8 • **G15** • **G20**	21.7Å	7.7Å	21.7Å	8.9Å
**G4** • **G7** • **G16** • **G19**	18.2Å	14.4Å	19.0Å	14.6Å

*As for the first G base at the 5' end, G1, misses the phosphate atom, which is replaced by C5' atom in calculation of the average groove width.

Table S3. The unimolecular G4s contains four G-tetrads in Protein Data Bank (PDB) up to 09 July 2025. The only RNA sequence is colored in yellow. The left-handed and right-left hybrid, in which one two-layered G4 block is right-handed and the other two-layered G4 block is left-handed, parallel G4s are colored in light green.

	PDB ID	Sequence	G-tetrad	Topology	Method	Solution condition
1	201D	d[G4(T4G4)3]	*syn·syn·anti·anti*	***Antiparallel basket***	NMR	Na^+, pH 6.8
2	230D	d(G4TUTUG4T4G4UUTTG3I)	*syn·syn·anti·anti*	***Antiparallel basket***	NMR	Na^+, pH 6.0
3	2M6W	d(G4TTG4T4G4A2G4)	*syn·syn·anti·anti*	***Antiparallel basket***	NMR	Na^+, pH 6.8
4	5J6U	d(G4TTTG4T4G4A2G4)	*syn·syn·anti·anti*	***Antiparallel basket***	NMR	Na^+, pH 6.8
5	6FTU	d(G4GAG4TACAG4TACAG4)	*syn·syn·anti·anti*	***Antiparallel basket***	X-ray	K^+, pH 6.5
6	2N3M	d[T(GGT)$_3$TGTTG(TGG)$_3$TGGT]	*anti·anti·anti·anti*	***Parallel***	NMR	K^+, pH 7.0
7	6W9P<br>7JKU<br>7LL0	d(GTTG$_4$TTG$_4$GTTG$_4$TTG$_4$T)	*anti·anti·anti·anti*	***Parallel***	X-ray	K^+, pH 6.5
8	6XT7	d(GTTG$_4$TTG$_4$GTTG$_4$TTG$_4$)	*syn·anti·anti·anti/ syn·syn·syn·anti*	***Hybrid***	X-ray	K^+, pH 7.2
9	6K84	r[(GGA)$_4$A(GGA)$_4$]	*anti·anti·anti·anti*	***Parallel***	NMR	K^+, pH 6.2
10	2MS9<br>4U5M	d[T(GGT)$_3$GGTTG(TGG)$_3$TGTT]	*syn·anti·anti·anti/ anti·anti·anti·anti*	Left-handed ***parallel***	NMR<br>X-ray	K^+, pH 7.0
11	6GZ6	d[GT(GGT)$_3$GTTGT(GGT)$_3$GT]	*anti·anti·anti·anti*	Left-handed ***paralle***l	X-ray	K^+, pH 6.6
12	7DFY	d[(GGT)$_3$GTGTT(GGT)$_3$GTG]	*anti·anti·anti·anti*	Left-handed ***parallel***	X-ray	K^+, pH 6.0
13	7D5D	d[GGTGTGTGGTGGTGT(GGT)$_3$G]	*anti·anti·anti·anti*	Left-handed parallel	X-ray	K^+, pH 7.0
14	7D5E	d[GGTGTGTGTGTGGTGT(GGT)$_3$G]	*anti·anti·anti·anti*	Left-handed ***parallel***	X-ray	K^+, pH 7.0
15	7D5F	d[GGTGTGTGTGTGTGTGT(GGT)$_3$G]	*anti·anti·anti·anti*	Left-handed ***parallel***	NMR	K^+, pH 7.0
16	6JCE	d[G2T2G2TGTG2T2G2TTGT(GGT)$_3$G]	*anti·anti·anti·anti*	Right-left hybrid ***parallel***	NMR	K^+, pH 7.0
17	6QJO	d[G2T2G2TGTG2T2G2TGT(GGT)$_3$G]	*anti·anti·anti·anti*	Right-left hybrid ***parallel***	X-ray	K^+, pH 7.0
18	9CIY*	d[GT(GGT)$_3$GTT(GGT)$_3$GTG]	*anti·anti·anti·anti*	Right-left hybrid ***parallel***	X-ray	K^+, pH 6.5

*Released on 09 July 2025 in PDB, to be published.

Reference

1. Lavery, R., Moakher, M., Maddocks, J.H., Petkeviciute, D. and Zakrzewska, K. (2009) Conformational analysis of nucleic acids revisited: Curves+. *Nucleic Acids Res*, **37**, 5917-5929.
